# Performance Evaluation and Field Validation of Next-Generation QMEMS Accelerometers for Seismology, Structural Health Monitoring, and Impact-Based Earthquake Early Warning

**DOI:** 10.3390/s26175528

**Published:** 2026-08-31

**Authors:** Domenico Patanè, Masayoshi Todorokihara, Gioacchino Fertitta, Claudio Martino, Giuseppe Occhipinti, Antonino Sicali, Francesco Sabella

**Affiliations:** 1 Istituto Nazionale di Geofisica e Vulcanologia, Sezione di Catania-Osservatorio Etneo, 95125 Catania, Italy; gioacchino.fertitta@ingv.it (G.F.); antonino.sicali@ingv.it (A.S.); 2 Centro “Monitoraggio Sismico Urbano e delle Strutture” INGV, 95125 Catania, Italy; claudio.martino@ingv.it (C.M.); inggiuseppeocchipinti@gmail.com (G.O.); 3 MD Products Development Department Microdevices Operations Division Fujimi Plant, Seiko Epson Corporation, 281 Fujimi, Fujimi-machi 399-0293, Suwa-gun, Nagano, Japan; todorokihara.masayoshi@exc.epson.co.jp; 4 Istituto Nazionale di Geofisica e Vulcanologia—Osservatorio Vesuviano, 80124 Napoli, Italy; 5 Epson Europe Electronics GmbH, Riesstrasse 15, 80992 Munich, Germany; francesco.sabella@epson-electronics.de

**Keywords:** QMEMS accelerometers, Smart Sensor Box, Urban Seismic Observatory, Structural Health Monitoring, Operational Modal Analysis, impact-based EEW

## Abstract

Recent advances in Micro-Electro-Mechanical Systems (MEMSs) have enabled the development of accelerometers increasingly suitable for seismological and structural engineering applications. Quartz MEMS (QMEMS) sensors combine low self-noise, wide dynamic range, excellent thermal stability, and compact dimensions, providing a cost-effective alternative to conventional force-balance and piezoelectric accelerometers. This study presents the development and validation of a complete QMEMS-based sensing platform for seismic monitoring and Structural Health Monitoring (SHM), integrating the recently introduced Epson M-A370 accelerometer, a Smart Sensor Box with precise timing synchronization, and embedded acquisition and edge-processing capabilities. The platform was evaluated through comprehensive laboratory and field experiments. The M-A370 was experimentally compared with the M-A352 and a reference force-balance accelerometer, while complementary M-A352 tests included representative MEMS and piezoelectric accelerometers. The experimental results indicate that accelerometer self-noise is a primary factor governing the reliability of Operational Modal Analysis (OMA) and long-term SHM. Self-noise densities below 1 μg/√Hz, preferably below 0.5 μg/√Hz, and, for the most demanding applications, approaching or below 0.1 μg/√Hz, represent practical performance targets for robust modal identification and reliable tracking of structural dynamic properties under weak ambient excitation or in very quiet environments. These values, however, are not exclusion thresholds, as higher-noise accelerometers can reliably record ground motions sufficiently above their instrumental noise floor. The ultra-low-noise M-A370 (0.02 μg/√Hz) delivers data quality comparable to engineering-grade force-balance accelerometers. The proposed platform, combining ultra-low-noise QMEMS technology, precise timing synchronization, and embedded processing, provides a scalable framework for Urban Seismic Observatories, distributed SHM, OMA, and impact-based Earthquake Early Warning (EEW) across buildings, bridges, and heritage structures.

## 1. Introduction

Accelerometric measurements play a fundamental role in both seismology and structural engineering, providing direct observations of ground and structural motion over a wide dynamic range, from weak ambient vibrations to strong earthquake shaking. In seismological applications, accelerometers are particularly important for near-field monitoring and engineering seismology, where large-amplitude motions must be recorded without clipping while preserving waveform fidelity and timing accuracy. These recordings are routinely used to derive engineering ground-motion parameters and intensity measures, including Peak Ground Acceleration (PGA), Peak Ground Velocity (PGV), Peak Ground Displacement (PGD), Arias Intensity, and response spectra [1,2]. These measures constitute the basis for seismic hazard assessment, ShakeMap generation, rapid impact assessment, and modern Earthquake Early Warning (EEW) systems [3,4,5]. Beyond peak quantities such as PGA, PGV, and PGD, energy- and duration-based intensity measures derived from acceleration records are increasingly adopted to characterize earthquake shaking, evaluate structural response, and assess structural damage, particularly within performance-based earthquake engineering frameworks [2,6,7,8].

At the urban scale, recent advances in compact digital instrumentation and sensing technologies have promoted the development of Urban Seismic Networks (USNs) and Urban Seismic Observatories (USOs), designed to characterize spatial variations in ground motion with high resolution and to support rapid impact assessment and emerging impact-based Earthquake Early Warning (EEW) approaches. In Italy, representative examples include the Urban Seismic Observatory of Catania [9] and the more recent urban monitoring networks deployed in the Campi Flegrei, Messina–Reggio Calabria, and Potenza.

Over the past two decades, similar initiatives have been established worldwide through the GeoNet strong-motion infrastructure in New Zealand [10,11], the K-NET and KiK-net networks in Japan [12], the metropolitan monitoring systems operated by the China Earthquake Administration [13,14], the SASMEX framework in Mexico [5,15], and the Community Seismic Network in the United States [16,17]. Together, these initiatives illustrate the global transition toward increasingly dense and real-time seismic monitoring, particularly in urban areas where high-resolution observations are essential for scientific investigations, engineering applications, and rapid impact assessment.

Accelerometers are equally important in structural engineering, where they are deployed on buildings, bridges, towers, cultural heritage structures, and critical infrastructures to record ambient and earthquake-induced vibrations. Such measurements form the basis for Operational Modal Analysis (OMA) and Structural Health Monitoring (SHM), enabling the estimation and long-term tracking of modal parameters, including natural frequencies, damping ratios, and mode shapes. Temporal variations in these modal parameters may indicate changes in structural properties and therefore provide vibration-based indicators for damage detection and structural integrity assessment [18,19,20,21].

Because many civil structures exhibit low-frequency vibration modes excited by extremely weak ambient motions, SHM applications impose stringent requirements on sensor performance, including ultra-low self-noise, excellent low-frequency resolution, long-term stability, and precise timing synchronization among distributed sensing nodes [20,21,22,23]. Traditionally, these applications have relied on high-performance force-balance accelerometers and conventional piezoelectric sensing technologies [23,24]. While both technologies provide excellent metrological performance, they generally involve higher costs, installation complexity, and maintenance requirements. Although these instruments remain reference solutions for many scientific and engineering applications, their widespread deployment in dense urban networks and distributed SHM systems is frequently constrained by economic and logistical considerations.

Recent advances in Micro-Electro-Mechanical Systems (MEMS) technology are progressively reshaping both seismological monitoring [9,25] and Structural Health Monitoring [18,20] by enabling compact, low-power, and cost-effective sensing systems suitable for large-scale deployments. Over the past decade, low-noise MEMS accelerometers have emerged as a promising alternative for Urban Seismic Networks, OMA, distributed SHM systems, and edge-based Earthquake Early Warning architectures. Nevertheless, many conventional MEMS devices still exhibit limitations related to self-noise, thermal drift, low-frequency response, and long-term stability, particularly when employed for weak-motion monitoring and modal identification tasks.

Among the various MEMS technologies currently available, resonant Quartz MEMS (QMEMS) devices have recently attracted considerable attention because they combine the long-term stability and high mechanical quality factor of quartz resonators with highly integrated MEMS fabrication processes [26], thus representing a significant evolution in MEMS accelerometer technology. Unlike conventional capacitive MEMS devices, they employ resonant quartz elements as acceleration-to-frequency transducers, exploiting the scalability and compactness of MEMS fabrication while benefiting from the thermal stability, low hysteresis, and intrinsic frequency stability of quartz resonators. As a result, QMEMS accelerometers offer ultra-low self-noise, high linearity, wide dynamic range, and excellent long-term stability, making them particularly well suited for weak- and strong-motion seismology, OMA and SHM. However, despite these favorable characteristics, the metrological performance of the sensing system alone does not guarantee reliable OMA or effective SHM. Besides sensor performance, the quality of modal identification and long-term SHM depends on the overall monitoring strategy, including the preliminary dynamic characterization of the structure, the definition of monitoring objectives, the selection and placement of sensors, and the adopted signal-processing and modal identification procedures. Consequently, sensor layout represents a key component of SHM system design, with increasing attention being devoted in the recent literature to monitoring strategies and optimal sensor placement [27].

Against this background, the present work provides a comprehensive experimental evaluation and field validation of the QMEMS sensing units and the Smart Sensor Box acquisition system, with particular emphasis on the recently introduced Epson M-A370 accelerometer, released in 2025 as the latest evolution of the M-A352 sensor. The study includes laboratory characterization of the QMEMS accelerometers, evaluation of the Smart Sensor Box timing performance under GNSS-disciplined and GNSS-denied conditions, and experimental assessment of a temperature-compensated VCXO (TCVCXO) for enhanced holdover performance. These original contributions are complemented by the first field validation of the M-A370 at Durham Castle and by an additional deployment on a slender steel pedestrian bridge for ambient vibration measurements and OMA. Furthermore, Section 4 provides an overview of representative applications of QMEMS technology in seismology, Structural Health Monitoring (SHM), Operational Modal Analysis (OMA), and impact-based Earthquake Early Warning (EEW), drawing on the experience gained over recent years. Although some deployments build upon previous studies, this is the first work to integrate laboratory characterization, timing validation, and multi-scale field applications within a unified assessment of the QMEMS-based sensing platform.

## 2. Accelerometric Sensors, Acquisition System, and Experimental Procedures

Recent advances in accelerometric technologies have transformed both seismic monitoring and Structural Health Monitoring (SHM), enabling the transition from conventional force-balance and piezoelectric instruments to highly integrated MEMS-based solutions.

Modern applications increasingly require sensors capable of simultaneously resolving weak ambient vibrations and strong earthquake motions while satisfying stringent requirements in terms of self-noise, dynamic range, bandwidth, timing synchronization, long-term stability, and deployment scalability. These requirements are particularly critical for Operation Modal Analysis (OMA), distributed Structural Health Monitoring (SHM) systems, Urban Seismic Observatories, and emerging impact-based EEW applications. Within this context, Quartz MEMS (QMEMS) technology represents a significant evolution of microfabricated accelerometers, combining the scalability and low power consumption of MEMS devices with the excellent thermal stability and low-noise characteristics of quartz resonators. The following sections describe the accelerometers and reference instrumentation considered in this study, the multiparametric acquisition architecture specifically developed for seismic and SHM applications, and the laboratory and field experimental procedures adopted to evaluate sensor performance and representative application scenarios.

### 2.1. QMEMS Technology and Reference Instrumentation

To provide a benchmark for evaluating the performance of the investigated QMEMS sensors, several representative MEMS, QMEMS, force-balance, and Integrated Electronics Piezo-Electric (IEPE) accelerometers commonly adopted in seismic and structural monitoring applications were considered.

Force-balance accelerometers (FBAs) have long represented the reference technology for strong-motion seismology, engineering seismology, and high-performance vibration monitoring, including OMA. By employing closed-loop servo mechanisms that maintain the proof mass near its equilibrium position, these instruments allow excellent linearity, stable low-frequency response, wide dynamic range, and very low self-noise [28]. Consequently, they are widely adopted in national and regional seismic networks, high-performance structural monitoring, and qualification procedures for strong-motion instrumentation [28,29]. Within the framework established by the Advanced National Seismic System (ANSS), Class-A strong-motion accelerometric systems are characterized by very high useful resolution and dynamic range, typically exceeding 110 dB [29].

Piezoelectric accelerometers, including IEPE devices, are also widely employed in vibration testing, OMA, and industrial monitoring owing to their robustness, high sensitivity, low noise, and excellent high-frequency response [30,31]. However, because of their intrinsically AC-coupled nature, they do not offer a true DC response and are therefore less suitable for very-low-frequency and quasi-static measurements [32]. In addition, wiring requirements, power consumption, and installation complexity may limit their applicability in dense and distributed monitoring networks. For these reasons, increasing attention has recently been devoted to low-noise MEMS and QMEMS technologies, which combine DC response, low power consumption, compact size, and ease of integration with embedded processing and wireless communication systems.

The rapid development of Micro-Electro-Mechanical Systems (MEMS) technology has introduced compact and cost-effective accelerometers suitable for different applications and large-scale deployments. Capacitive MEMS sensors estimate acceleration through displacement-induced variations in differential capacitance [25], whereas piezoresistive MEMS devices exploit strain-induced resistance variations and are commonly employed in high-g and shock applications [33]. Their capability to enable response down to DC makes MEMS accelerometers particularly attractive for dense Urban Seismic Networks and SHM systems [17,22,34].

**Figure 1:**
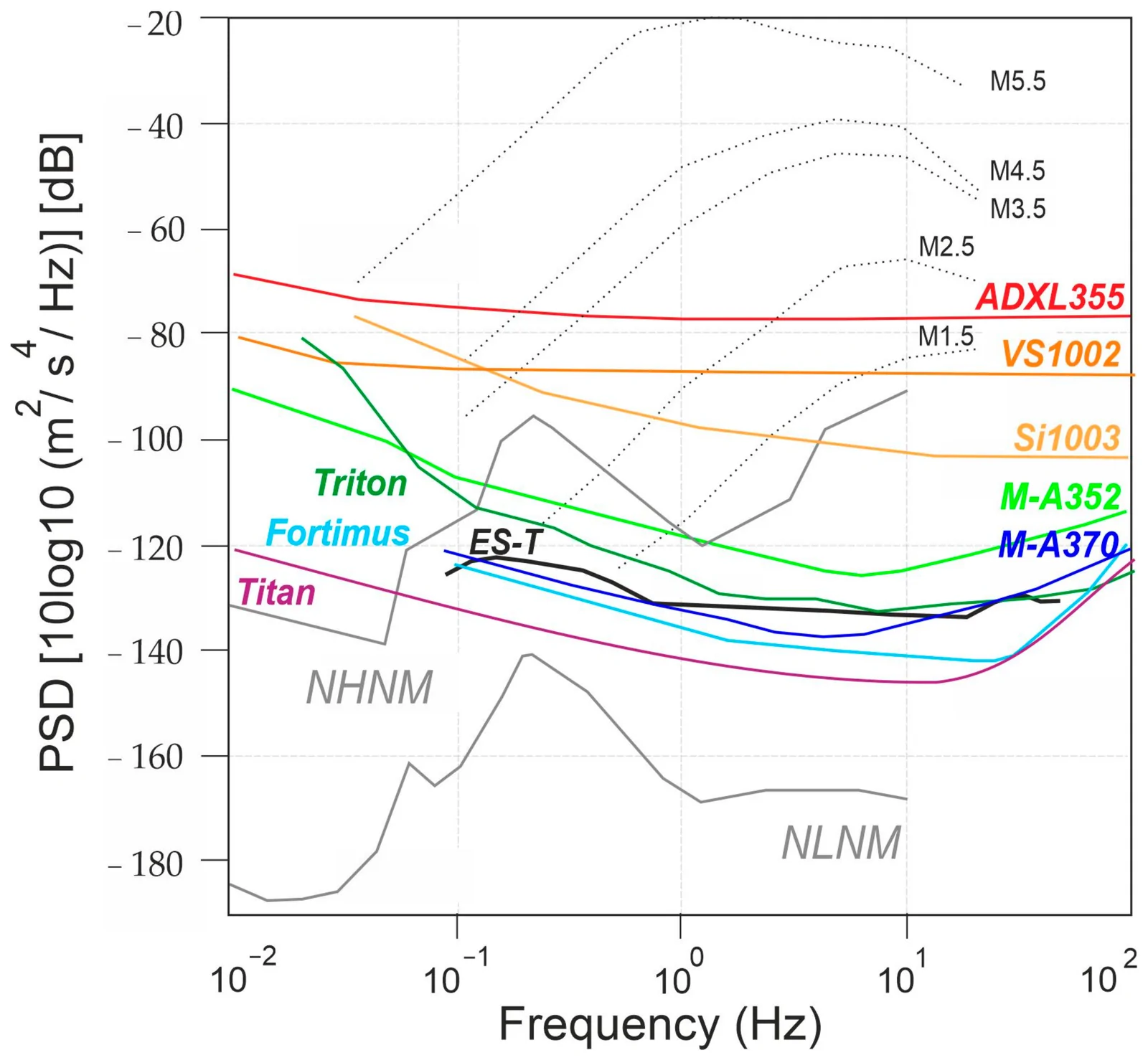
Comparison of the manufacturer-specified self-noise power spectral density (PSD) of representative accelerometer technologies used in seismic monitoring and Structural Health Monitoring (SHM) applications. The comparison includes conventional MEMS accelerometers (ADXL355, VS1002, and SI1003), QMEMS accelerometers (Epson M-A352 and M-A370), and force-balance accelerometers (Nanometrics Titan, Güralp Fortimus, Kinemetrics EpiSensor ES-T, and Lunitek Triton). The New High Noise Model (NHNM) and New Low Noise Model (NLNM) [35], together with reference spectra (thick black curves) for earthquakes of increasing magnitude (M 1.5–M 5.5) at an epicentral distance of 10 km (dashed gray curves), are included for comparison. The PSD curves were derived from the self-noise specifications reported in the corresponding manufacturers’ datasheets.

Table 1 and Table 2 summarize the principal characteristics of the accelerometers considered in this study. Several of these instruments were experimentally evaluated or deployed in the laboratory and field case studies presented in this work, covering both seismic monitoring and structural engineering applications. SI units (m/s^2^) are used for laboratory characterization and quantitative analyses, whereas equivalent values in g are also reported for engineering and strong-motion applications (1 g = 9.80665 m/s^2^). Sensor self-noise is reported either as power spectral density (PSD) or as noise density (μg/√Hz), according to the convention adopted by the corresponding manufacturer or analysis. For consistency with the PSD values reported in Figure 1, acceleration noise density (a_n_, in μg/√Hz) can be converted into acceleration PSD (expressed as PSD [10 ⋅log10 (m^2^/s^4^/Hz)] [dB]) according to PSD = 10·log10[(a_n_ × 9.80665 × 10^−6^)^2^].

Figure 1 summarizes the manufacturer-specified self-noise power spectral density (PSD) of a representative selection of these accelerometers together with selected force-balance instruments against ambient seismic noise models and representative earthquake spectra at an epicentral distance of 10 km.

The comparison highlights that, despite their advantages in terms of cost and scalability, conventional MEMS accelerometers generally exhibit higher self-noise than force-balance accelerometers and velocimetric seismometers, providing the context for the practical self-noise targets discussed below. This becomes particularly relevant for weak-motion seismology, OMA, and long-term SHM, where the ability to resolve signals close to the ambient noise level is essential.

Accordingly, sensor self-noise is regarded as one of the most critical performance parameters of seismic accelerometers, since the ability to resolve low-amplitude ground and structural vibrations is ultimately controlled by the relationship between instrument self-noise and the ambient vibration level. Although no universally accepted self-noise limits exist, indicative engineering targets can be derived from published studies on seismic background noise and accelerometer performance [24,35], together with the experimental evidence considered in this work.

In general, self-noise values below approximately 1 µg/√Hz represent a practical target for a broad range of weak-motion seismic and structural-monitoring applications, whereas values below approximately 0.5 µg/√Hz are preferable for low-amplitude ambient-vibration measurements and reliable OMA/SHM applications. For particularly demanding measurements in very quiet environments, values approaching or below 0.1 µg/√Hz are desirable. These values should therefore be regarded as indicative engineering guidelines rather than universal acceptance criteria.

**Figure 2:**
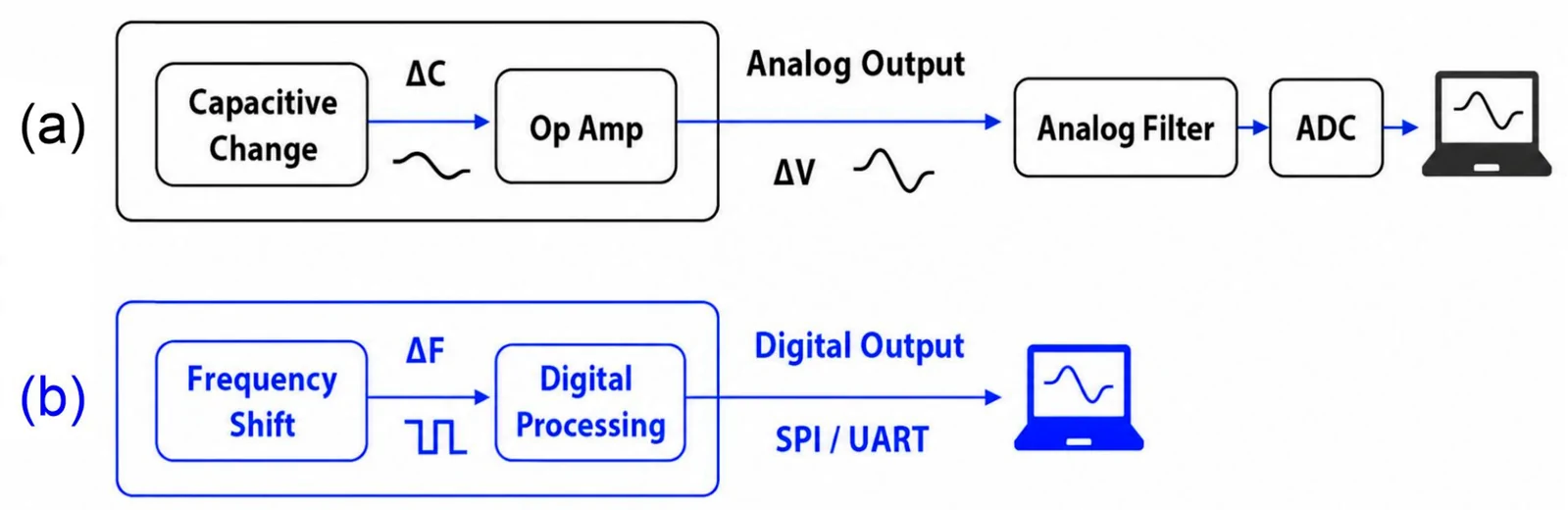
Comparison of signal processing chains between a conventional capacitive MEMS accelerometer (**a**) and the QMEMS accelerometer (**b**). The QMEMS approach utilizes direct acceleration-to-frequency conversion and digital frequency measurement, avoiding the conventional analog amplification and voltage-based A/D conversion chain, thereby reducing the associated sources of noise and non-linearity.

The substantial differences in self-noise performance among the accelerometers summarized in Figure 1 can be better understood by considering the fundamentally different sensing principles adopted by conventional capacitive MEMS and quartz-resonant MEMS technologies, schematically illustrated in Figure 2. In conventional capacitive MEMS sensors, acceleration is measured through displacement-induced variations in differential capacitance, which are subsequently converted into electrical signals. As illustrated in the upper part of Figure 2, this measurement chain relies on analog signal conditioning and analog-to-digital conversion stages that inherently introduce trade-offs among resolution, dynamic range, and linearity. Furthermore, achieving stable low-frequency performance, particularly from DC to a few hertz, remains difficult because of 1/f noise, thermal drift, and long-term instabilities affecting both the sensing element and the analog front-end.

Quartz-resonant MEMS (QMEMS) accelerometers adopt a fundamentally different sensing principle, illustrated in the lower part of Figure 2. Instead of directly measuring proof-mass displacement, these devices employ a Double-Ended Tuning Fork (DETF) quartz resonator as the sensing element. Acceleration applied to the proof mass generates axial stress within the resonator, producing a measurable shift in its resonant frequency.

**Figure 3:**
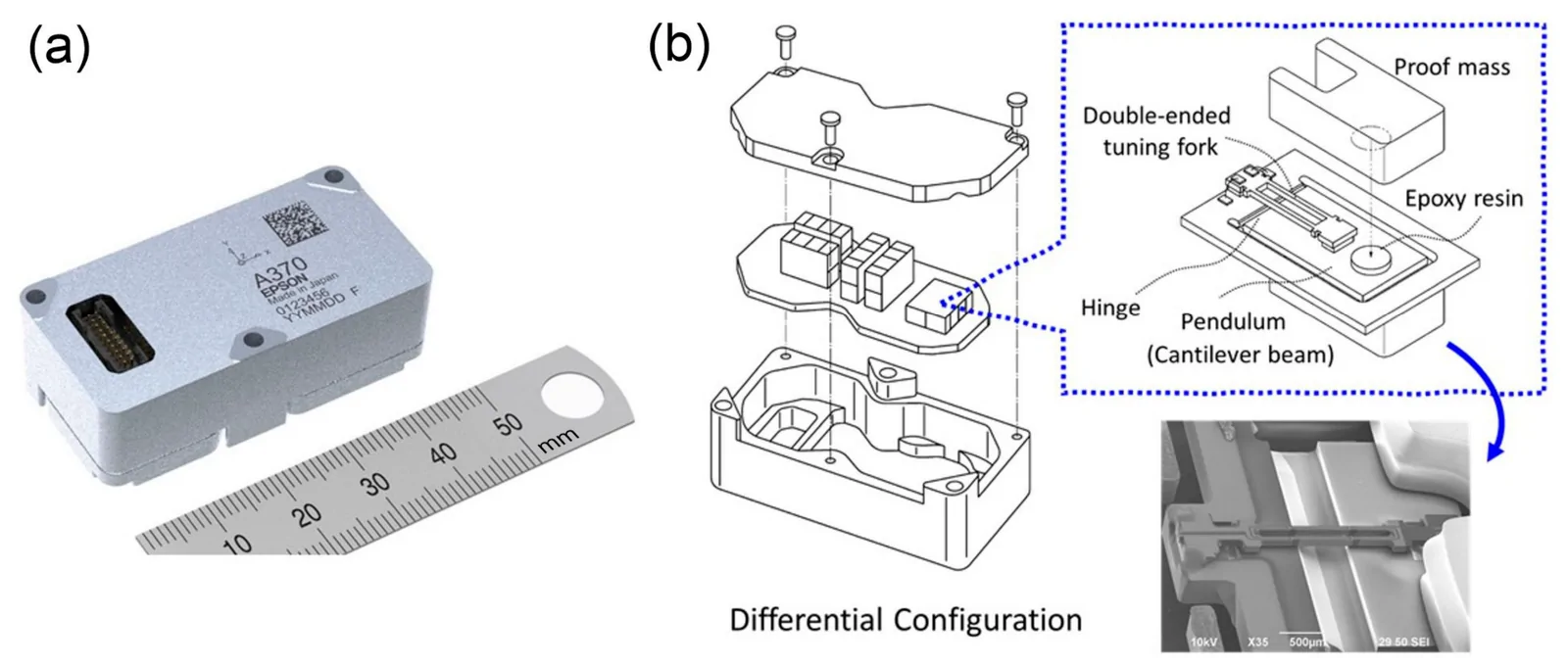
(**a**) External view of the M-A370 accelerometer module. The compact aluminum housing (48 × 24 × 16 mm^3^) contains the 3-axis sensor assembly and digital readout electronics, offering a significant size advantage for dense array deployments. (**b**) Internal structure of the M-A370 sensor module. The exploded view (left) shows the differential arrangement of the six transducer units (two per axis) mounted within the package. The detailed schematic (top right) illustrates the high-density tungsten proof mass attached to the quartz cantilever and DETF resonator. The SEM image (bottom right) shows the fabricated Double-Ended Tuning Fork (DETF) resonator.

This acceleration-to-frequency conversion approach provides a highly linear output that can be processed digitally with very high precision, reducing the influence of analog conditioning electronics and minimizing quantization and linearity limitations commonly associated with conventional readout chains [36]. In this configuration, the achievable dynamic range is primarily governed by the frequency measurement system rather than by the analog conversion stage. In the following, we describe the characteristics of the Epson M-A370 QMEMS accelerometer (Figure 3a), a new-generation device developed as an evolution of the widely adopted M-A352 platform [36].

According to design information provided by the manufacturer, one of the principal factors contributing to the enhanced sensitivity of the M-A370 is the adoption of a high-density tungsten proof mass, replacing the phosphor-bronze mass employed in the previous generation. As shown in Figure 3b, this design modification substantially increases inertial mass while preserving the compact dimensions of the sensor. Combined with an optimized DETF geometry derived through finite-element modeling, this configuration increases acceleration sensitivity from approximately 152 Hz/g in the M-A352 to approximately 550 Hz/g in the M-A370. Such an improvement enhances the capability of resolving extremely weak acceleration signals associated with microtremors, ambient vibrations, and weak-motion seismic recordings.

To exploit this increased sensitivity while maintaining robustness under field conditions, the M-A370 adopts a differential architecture consisting of six DETF resonators arranged as three independent pairs, one for each measurement axis (Figure 3b). In contrast to previous single-ended implementations, the differential configuration measures the frequency difference between paired resonators operating in a push-pull mode. This approach suppresses common-mode effects associated with temperature variations, packaging-induced stresses, and other environmental perturbations, resulting in improved bias stability and scale-factor consistency over a broad operating temperature range.

Further improvements are achieved through a fully digital frequency-measurement architecture based on a proprietary phase-measurement approach inspired by moiré-pattern sensitivity [36]. Implemented on a low-power FPGA platform, the system measures resonant-frequency variations with high precision while avoiding several sources of nonlinearity typically associated with conventional analog phase detectors and reciprocal frequency counters. Although the detailed proprietary implementation of the oscillation and frequency-measurement electronics cannot be disclosed, the approximately one-order-of-magnitude reduction in self-noise of the M-A370 relative to the previous-generation M-A352 can be interpreted as the combined effect of three principal factors: (i) the approximately 3.6-fold increase in transducer sensitivity resulting from the higher-density tungsten proof mass and optimized DETF geometry (152 Hz/g to approximately 550 Hz/g); (ii) the differential push-pull architecture, which provides an additional improvement through common-mode noise rejection; and (iii) the optimized oscillation and digital frequency-measurement circuitry [36]. Collectively, these design improvements enable the M-A370 to achieve a noise density of approximately 0.02 µg/√Hz in the 1–10 Hz frequency band (Table 1). Such performance approaches the levels traditionally associated with high-end force-balance accelerometers (Figure 1) and enables applications that have historically required more expensive instrumentation and, in some cases, also velocimeters.

These characteristics are particularly important for dense Urban Seismic Networks and distributed Structural Health Monitoring (SHM) systems [9,37], where the capability to resolve signals close to the ambient noise level directly influences the quality of modal identification, ambient vibration analysis, and weak-earthquake recordings. More generally, the evolution from conventional MEMS architectures toward quartz-resonant technologies reflects the broader transition toward sensing platforms capable of combining the metrological performance traditionally associated with force-balance accelerometers with the scalability, compactness, and deployment flexibility required for next-generation seismic and structural monitoring systems. Fully exploiting these sensor capabilities, however, also requires an integrated acquisition platform providing high-resolution digitization, precise timing synchronization, embedded processing, and reliable communication. These aspects are addressed by the multiparametric Smart Sensor Box architecture described in the following section.

### 2.2. Multiparametric Smart Sensor Box and Timing Architecture

To evaluate the performance of the Epson M-A352 and M-A370 QMEMS accelerometers for seismic monitoring and Structural Health Monitoring (SHM) applications, a multiparametric Smart Sensor Box was developed by further extending a previously proposed architecture [38]. The updated platform, finalized in 2025 in collaboration with specialized industrial partners, was employed throughout the laboratory investigations presented in this work.

**Figure 4:**
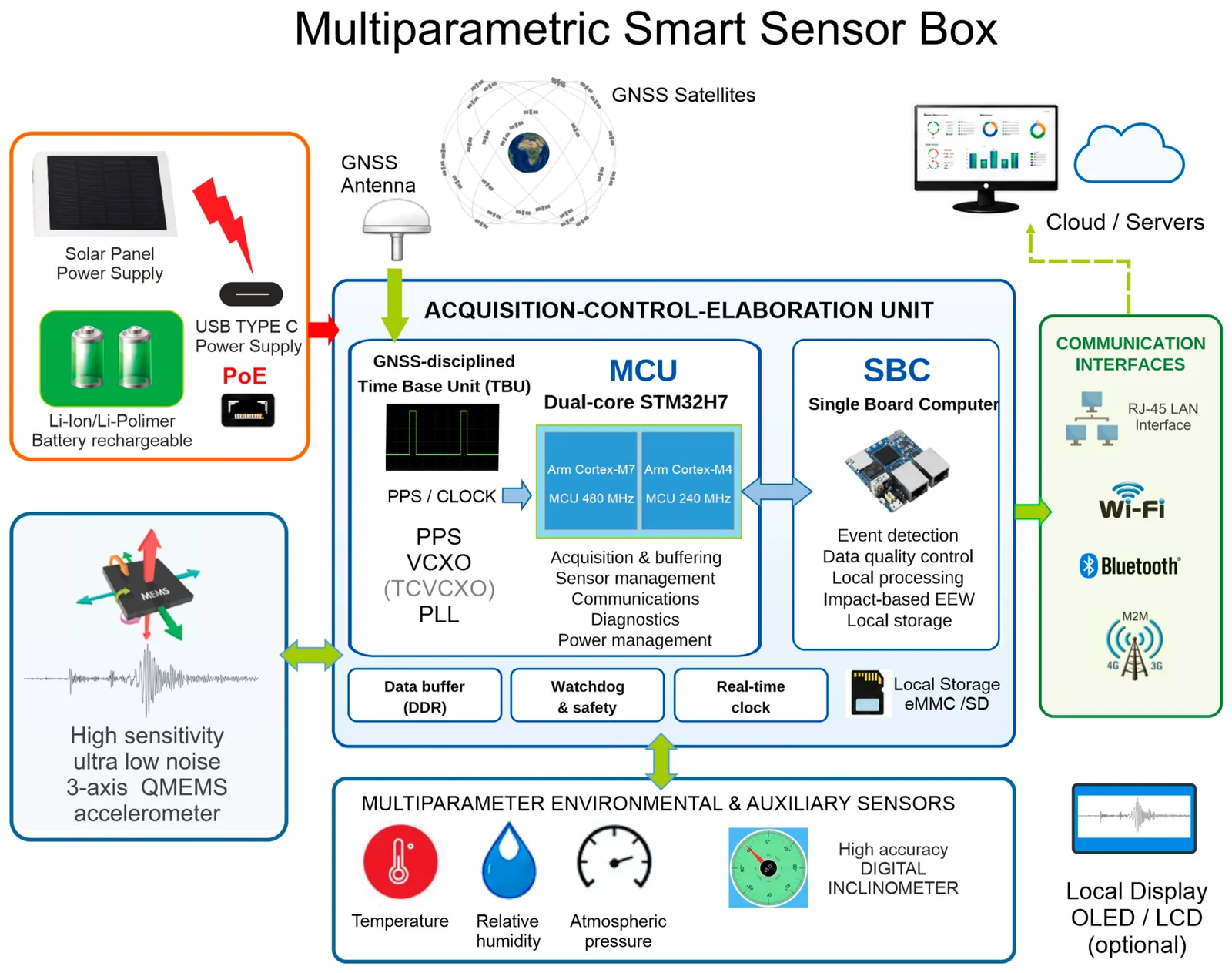
Functional architecture of the multiparametric Smart Sensor Box used in this study. The system integrates a high-sensitivity Epson M-A352/M-A370 QMEMS accelerometer, a GNSS-disciplined Time Base Unit (TBU), a dual-core STM32H7 microcontroller, and an embedded single-board computer providing edge-computing capabilities. The modular architecture supports deterministic timing, local data processing, multi-parameter environmental sensing, and distributed communication through wired and wireless interfaces, providing a scalable sensing framework for seismic monitoring and Structural Health Monitoring applications.

The Smart Sensor Box relies on a fully digital and modular acquisition architecture specifically conceived for seismic and SHM applications and supporting multi-parameter environmental monitoring. As illustrated in Figure 4, the system integrates high-sensitivity QMEMS accelerometers, a GNSS-disciplined Time Base Unit (TBU), a dual-core STM32H7 (STMicroelectronics, Geneva, Switzerland) microcontroller (MCU), an embedded Single Board Computer (SBC), communication interfaces, local storage capabilities, and auxiliary sensors. The architecture enables the integration of accelerometers, GNSS receivers, inclinometers, environmental sensors, and other digital sensing devices commonly adopted in distributed monitoring networks. Although the present study focuses on Epson QMEMS accelerometers, the acquisition, synchronization, and processing framework is sensor-independent and can be readily adapted to alternative sensing technologies.

Sensor acquisition, buffering, and communications are managed by the dual-core STM32H7 microcontroller, whereas timing synchronization is provided by the dedicated Time Base Unit. Building on the previously proposed architecture [38], the system separates high-rate dynamic measurements from environmental and diagnostic data streams, allowing vibration measurements to be acquired with deterministic timing and low jitter while independently collecting auxiliary parameters such as temperature, humidity, atmospheric pressure, inclination, power system information, and GNSS data. The timing subsystem is based on a dedicated Time Base Unit (TBU) incorporating a GNSS-disciplined oscillator, which provides a common and highly stable time reference, enabling all sensing nodes to remain accurately synchronized. Rather than distributing synchronization signals to remote sensors, the architecture ensures that all nodes operate with the same timing reference and maintain a high degree of temporal consistency. Such synchronization is essential for seismic monitoring, OMA, vibration-based damage detection, wavefield analysis, and other distributed measurement applications requiring sub-millisecond timing accuracy.

In addition to data acquisition and synchronization, the Smart Sensor Box combines the capabilities of the dual-core microcontroller with those of an embedded SBC, providing on-board signal processing, data-quality control, event detection, local data storage, and edge-computing capabilities. The SBC supplies the computational resources required for advanced functionalities, including impact-based EEW, strong-motion parameter estimation, local classification algorithms, and real-time generation of graphical products. Overall, this architecture provides a flexible, scalable, and sensor-independent platform for distributed seismic monitoring and SHM. Its modular design facilitates future hardware upgrades, the integration of additional sensing technologies, and the evolution toward Urban Seismic Observatories and advanced monitoring systems for critical infrastructures and cultural heritage assets.

### 2.3. Experimental Setups

Laboratory and field experiments were conducted to characterize the performance of the investigated sensors under conditions representative of both seismological and Structural Health Monitoring (SHM) applications.

The experimental campaign comprised laboratory calibration and dynamic shake-table tests, timing validation under both GNSS-disciplined and GNSS-denied conditions, and field deployments involving Urban Seismic Observatories, bridge monitoring, building monitoring, and cultural heritage structures. Laboratory experiments were performed at the L.E.D.A. (Laboratory of Earthquake Engineering and Dynamic Analysis) Research Centre of the University of Enna “Kore” using the SPEKTRA CS18P vibration calibration system (SPEKTRA Schwingungstechnik und Akustik GmbH Dresden, Dresden, Germany) equipped with an APS 129 electrodynamic shaker featuring hydrodynamic suspension and closed-loop motion control.

The calibration system supports secondary calibration procedures compliant with ISO 16063-21:2003 (Methods for the Calibration of Vibration and Shock Transducers—Part 21: Vibration Calibration by Comparison to a Reference Transducer. ISO: Geneva, Switzerland, 2003) and provides programmable, low-distortion sinusoidal excitation over a broad frequency and velocity range. Under these controlled laboratory conditions, the Epson M-A352 and M-A370 QMEMS accelerometers, together with a reference Güralp Fortimus force-balance accelerometer, were tested using identical excitation sequences to enable a direct comparison of their dynamic performance.

The laboratory characterization consisted of sinusoidal excitation tests covering frequencies between 0.5 and 100 Hz and nominal peak acceleration amplitudes ranging from 0.1 to 5 m s^−2^, according to the operating range of the investigated sensors and the capabilities of the calibration system. The recorded waveforms were analyzed using an automated processing workflow specifically developed for this study. The adopted signal-processing procedure is described in Section 3.1.

To complement the calibration experiments, the results of a comparative dynamic testing campaign performed on the 2:3 scale masonry building installed on one of the two large-scale shaking tables of the L.E.D.A. Research Centre are also presented [39]. The experimental facility enables controlled seismic excitation of large structural specimens under realistic dynamic loading conditions. During this experiment, a total of 34 sensors (92 channels), including Epson M-A352 QMEMS, Safran-Colibrys VS1002, Analog Devices ADXL355, and PCB Piezotronics 356A17 accelerometers, were deployed to investigate their performance and their capability to identify the modal properties of the structure.

Timing performance was evaluated through laboratory and field experiments involving pairs of co-located Smart Sensor Boxes operating under both GNSS-disciplined and GNSS-denied conditions. Oscilloscope measurements, phase analysis, coherence estimates, and sub-sample cross-correlation techniques were employed to quantify synchronization accuracy and relative timing stability. The adopted experimental procedures and the corresponding results are presented in Section 3.2.

Finally, several field deployments were carried out to validate the proposed sensing platform under real operating conditions. These included Urban Seismic Observatories in southern Italy, monitoring systems deployed on residential buildings in the Campi Flegrei area, continuous monitoring of the Ragusa Bridge, and permanent and temporary deployments on a cable-stayed steel pedestrian bridge in the UK, at the Bell Tower of the Basilica of Saint Francis in Assisi, and at Durham Castle, to support OMA investigations and long-term SHM. The latter two sites are UNESCO World Heritage sites. Together, these complementary laboratory and field experiments support the experimental basis for the performance assessment presented in Section 3 and for the representative applications discussed in Section 4.

## 3. Results

### 3.1. Laboratory Evaluation of Sensor Performance

**Figure 5:**
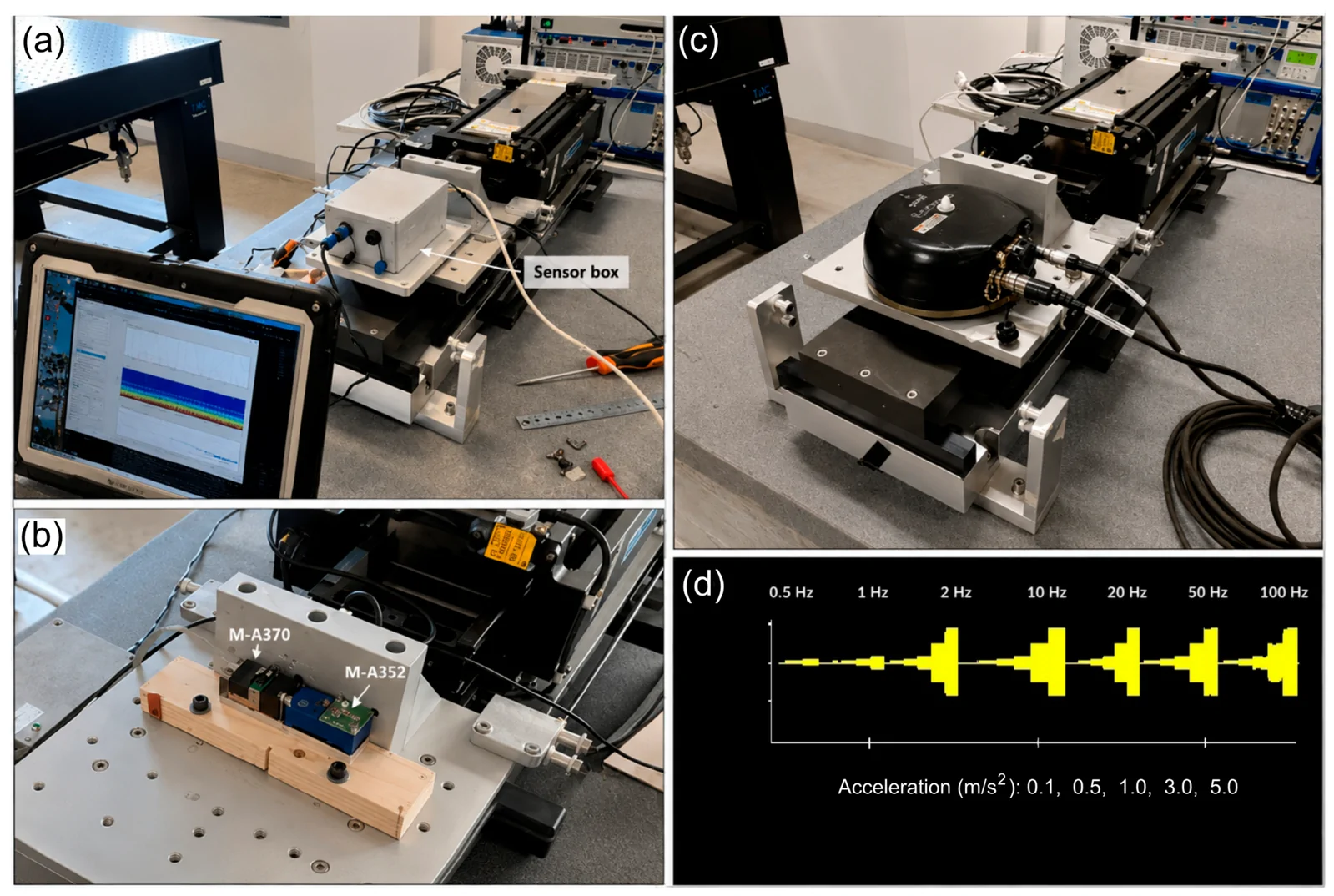
Experimental setup adopted for the laboratory validation of the proposed QMEMS-based acquisition systems at the L.E.D.A. (Laboratory of Earthquake Engineering and Dynamic Analysis) Research Centre. (**a**) Complete Smart Sensor Box mounted on the SPEKTRA APS129 electrodynamic shaker for testing under the operational configuration. (**b**) Epson M-A352 and M-A370 QMEMS sensing elements mounted side-by-side on a rigid fixture for direct comparison of the sensor response independently of the enclosure. (**c**) Güralp Fortimus force-balance accelerometer installed on the shaker table. (**d**) Frequency–amplitude excitation scheme adopted to evaluate sensor performance over the range of amplitudes and frequencies shown in the panel.

The laboratory validation presented in this section was designed to assess the amplitude accuracy and linearity of the proposed acquisition systems over the frequency range relevant to seismic monitoring and Structural Health Monitoring (SHM) applications. Figure 5 illustrates the three experimental configurations implemented using the APS 129-HF electrodynamic shaker. First, the complete Smart Sensor Boxes integrating the Epson M-A352 and M-A370 QMEMS accelerometers were tested in their operational configuration (Figure 5a). In the second setup (Figure 5b), the sensing elements alone were mounted directly on the shaker table and connected through shielded cables to the corresponding Smart Sensor Boxes positioned outside the shaker. This arrangement was adopted exclusively for the laboratory experiments to isolate the intrinsic response of the sensing elements from the possible mechanical influence of the enclosure. Both accelerometers were evaluated using the same acquisition electronics and an identical signal-processing chain, with all signals synchronously acquired at 500 samples per second (sps) using the sensors’ built-in 210 Hz low-pass filter. Finally, the Güralp Fortimus, which integrates a force-balance accelerometer, digitizer, and acquisition electronics into a single instrument, was mounted directly on the shaker table (Figure 5c). It was tested under the same set of excitation conditions and used as a performance benchmark for comparison with the QMEMS-based systems.

The experimental program comprised selected combinations of sinusoidal excitations at frequencies of 0.5, 1, 2, 10, 20, 50, and 100 Hz and nominal peak acceleration amplitudes of 0.1, 0.5, 1, 3, and 5 m s^−2^ (Figure 5d). These values define the overall test program rather than a complete factorial combination of all frequencies and amplitudes. The applied combinations were selected according to the force–displacement envelope of the APS 129-HF. In particular, testing at 0.5 Hz was limited to acceleration amplitudes of 0.1 and 0.5 m s^−2^, whereas higher amplitudes were applied only at compatible frequencies. For each implemented excitation condition, the calibration system generated the prescribed reference motion, while the outputs of the three acquisition systems were recorded for subsequent analysis.

The lower calibration limit of 0.5 Hz was primarily determined by the displacement constraints of the APS 129-HF shaker. According to the manufacturer’s datasheet, the shaker has a maximum stroke of 158 mm peak-to-peak (±79 mm), while its nominal operating range extends to DC (see the force–displacement performance envelope in the APS 129-HF datasheet: https://www.apsdynamics.com/product/aps-129; accessed on 20 July 2026. Since the displacement required for sinusoidal excitation increases as 1/f^2^, measurements at lower frequencies, for example, at 0.2 or 0.1 Hz, would require substantially larger displacement amplitudes. At 0.1 Hz, for example, the maximum theoretical acceleration corresponding to the full shaker stroke is only approximately 0.031 m/s^2^, well below the minimum test level of 0.1 m/s^2^ adopted in this study.

Beyond this displacement constraint, the larger platform excursions and lower target accelerations would increase sensitivity to parasitic motion, residual pitch and roll, and small platform inclinations that may project a component of gravitational acceleration onto the measurement axis. Payload effects and limitations of the control and reference measurement chain could introduce additional uncertainty. These combined factors would progressively narrow the achievable acceleration range and increase measurement uncertainty below 0.5 Hz, despite the reduced friction provided by the air-bearing suspension.

Although these calibration-system limitations prevented a reliable extension of the experimental characterization below 0.5 Hz using the adopted shaker configuration, the 0.1–0.5 Hz band remains particularly relevant for investigating the dynamic response of flexible and long-period structures, including tall buildings, long-span bridges, and seismically isolated structures. Nevertheless, the adopted 0.5–100 Hz calibration range encompasses the fundamental frequencies of many conventional low- to medium-rise buildings and other civil structures, whose principal modes generally occur above 0.5 Hz, and covers a broad spectrum of the seismic-monitoring and SHM applications considered in this study. Furthermore, although the shaker can reach higher acceleration levels, the present experimental program was limited to 5 m/s^2^ (approximately 0.5 g) to prioritize the assessment of sensor accuracy and linearity across a broad frequency range. However, further tests will be conducted at approximately 10 m/s^2^ (1 g) over the 2–30 Hz range and 15 m/s^2^ (1.5 g) over the more conservative 5–20 Hz range, consistent with the force–displacement envelope of the APS 129-HF shaker. These measurements will provide a more comprehensive assessment of sensor linearity and strong-motion performance.

Within the frequency–amplitude domain investigated in the present experimental program, all recorded waveforms were processed using an automated Python 3.11 workflow specifically developed for this study, ensuring a consistent, objective, and fully reproducible evaluation of sensor performance. Because each excitation record included transient phases associated with the stabilization of the vibration calibration system following changes in excitation frequency or amplitude, the first step consisted of identifying the stationary portion of the signal before quantitative analysis. Each waveform was divided into consecutive 10 s windows, which were mean-corrected, detrended, and tapered using a Gaussian window prior to frequency-domain analysis. Stable windows were automatically identified according to predefined frequency- and amplitude-consistency criteria, and the longest continuous stationary interval was retained for further processing.

The selected waveform was subsequently analyzed using a fixed-frequency least-squares sine fit to estimate the measured acceleration amplitude, excitation frequency, phase, and residual offset. The fully automated workflow ensured identical processing of all datasets without user intervention, thereby enabling an objective and unbiased comparison of the investigated acquisition systems.

**Figure 6:**
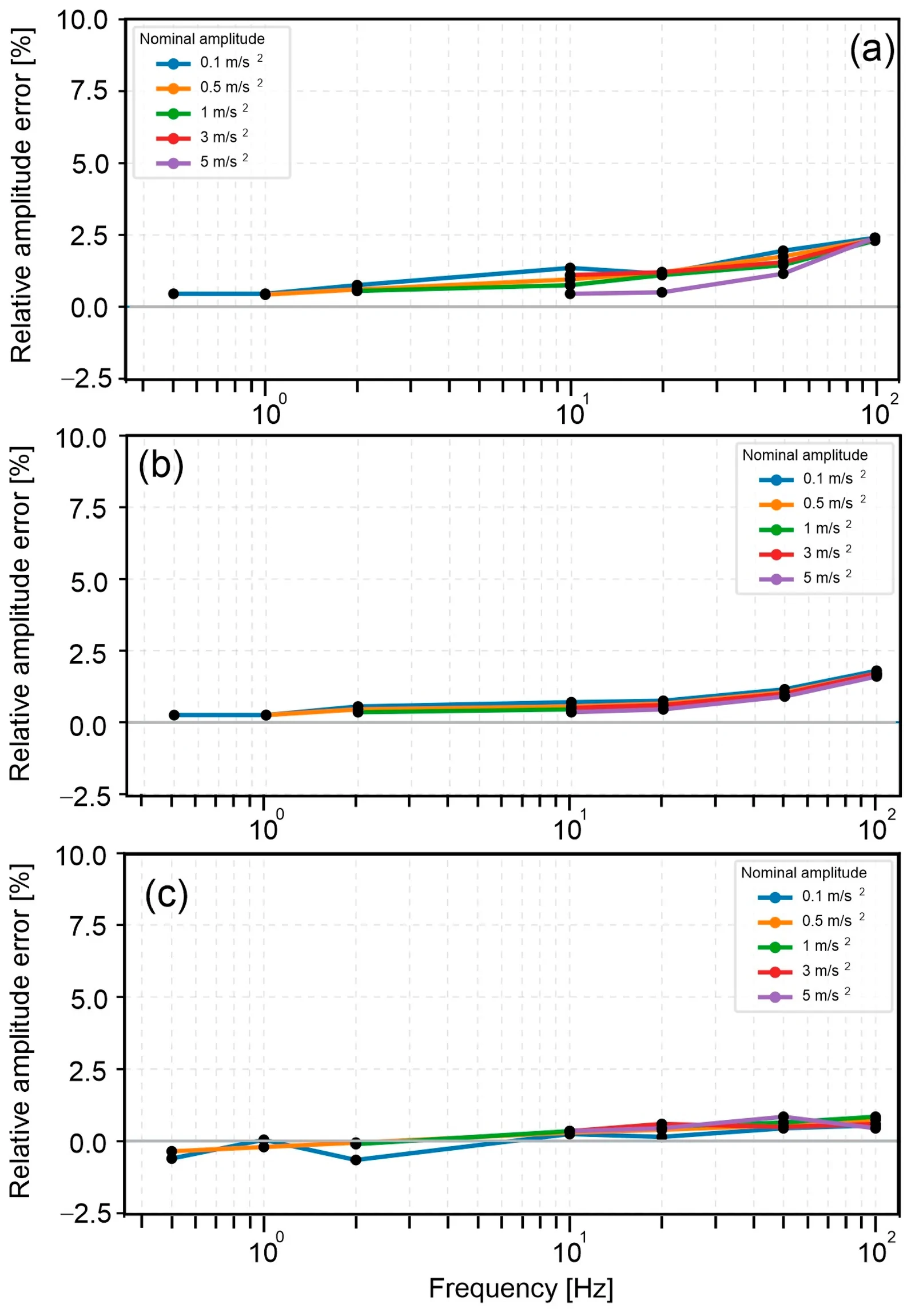
Measured relative amplitude error as a function of excitation frequency for the three configurations: (**a**) Sensor Box integrating the Epson M-A370 QMEMS; (**b**) standalone Epson M-A370 QMEMS accelerometer mounted directly on the shake table; and (**c**) Güralp Fortimus force-balance accelerometer. The curves correspond to sinusoidal excitation tests performed at nominal peak acceleration amplitudes of 0.1, 0.5, 1, 3, and 5 m s^−2^.

Figure 6a–c summarize the laboratory validation results obtained for the three configurations. Figure 6a,b present the results for the QMEMS M-A370 sensor. The corresponding results obtained for the M-A352 were nearly identical and are therefore omitted for brevity. All configurations accurately reproduce the applied sinusoidal motion over the investigated frequency range. The measured amplitudes remain in close agreement with the nominal excitation values, with deviations generally within the uncertainty limits of the calibration system. Moreover, no significant dependence of the amplitude error on the excitation level is observed, indicating that all acquisition systems preserve a linear response throughout the investigated dynamic range.

A modest increase in the measured amplitude error is, however, observed at the highest test frequencies, particularly approaching 100 Hz. Because a comparable trend is evident for all tested configurations, including the reference force-balance accelerometer, this behavior is unlikely to originate from the intrinsic response of the sensing elements. Instead, it is more plausibly associated with the experimental conditions, including the dynamic characteristics of the vibration calibration system, the mounting fixtures and sensor supports, as well as other sources of high-frequency mechanical disturbance within the laboratory environment.

Nevertheless, the observed deviations remain limited to a few percent and are therefore considered negligible for the intended applications.

Overall, excluding the expected limitations at the margins of the investigated frequency range, both QMEMS accelerometers exhibited excellent linearity and accuracy across the operational bandwidth relevant to seismic monitoring and SHM, whose frequency content is predominantly concentrated below 50 Hz. No significant differences were observed between the results obtained with the externally connected QMEMS sensors and the integrated sensor configuration. This confirms that integration of the sensing element into the acquisition unit can be achieved without degrading metrological performance, while providing the practical advantages of a more compact and robust architecture for field deployment and long-term monitoring. These results indicate that the tested QMEMS accelerometers and associated acquisition system achieve metrological performance comparable to that of the reference force-balance instrument over the frequency band relevant to seismic and structural engineering applications, supporting their use in both permanent seismic monitoring networks and research-grade structural monitoring systems.

**Figure 7:**
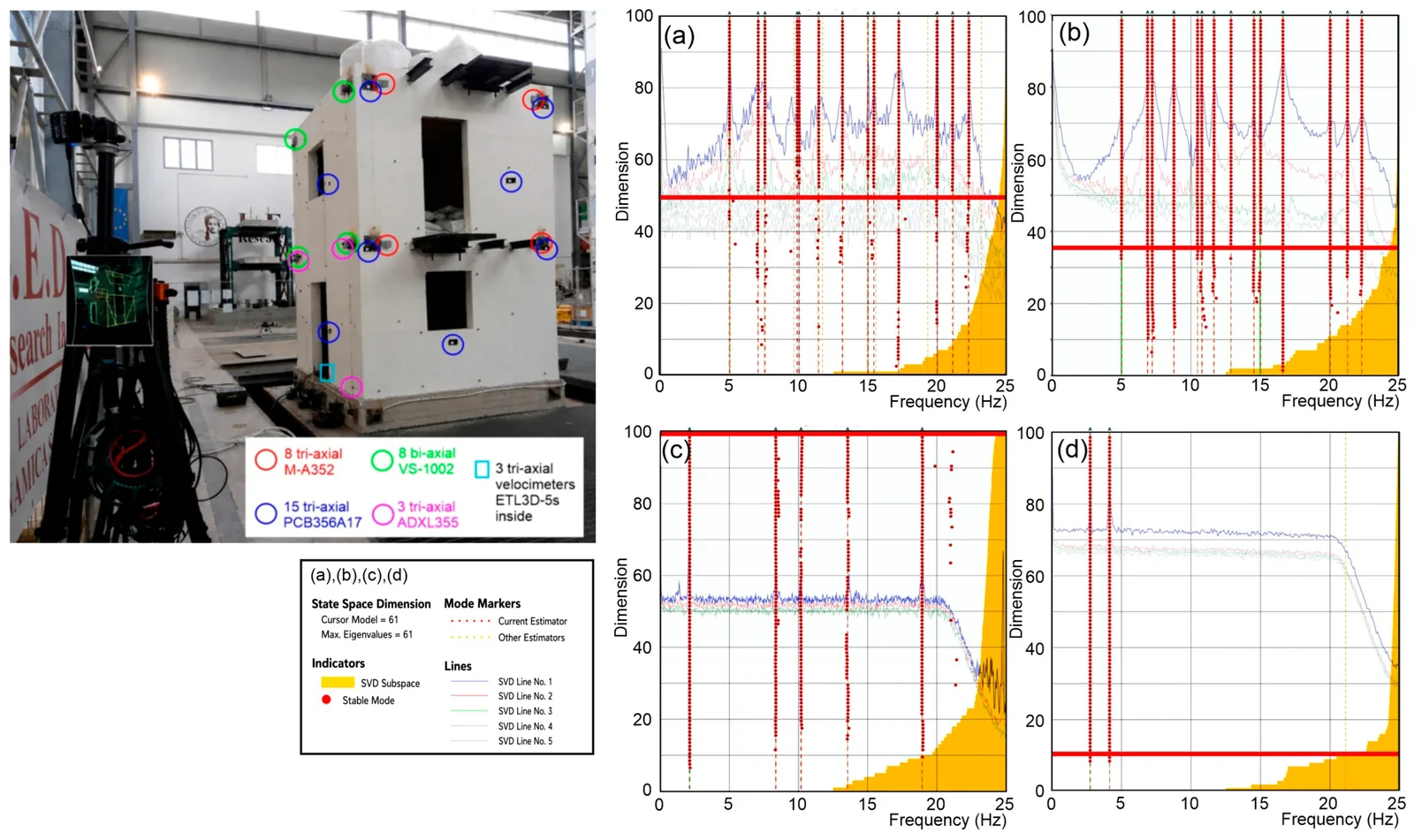
Experimental setup and representative stabilization diagrams obtained during the dynamic testing campaign on the 2:3 scale masonry building at the L.E.D.A. laboratory (rearranged from [39]). The comparison among the M-A352 (**a**), PCB 356A17 (**b**), VS1002 (**c**), and ADXL355 (**d**) sensors highlights the influence of the different overall noise levels of the sensing systems on the identification of weak modal responses, with the M-A352 and PCB 356A17 providing the clearest stabilization diagrams.

Beyond the laboratory characterization presented above, additional supporting evidence of the practical performance of the investigated sensing technologies is provided by the comparative shake-table experiments previously reported in [39]. In that study, a 2:3 scale masonry building installed on one of the two large-scale shaking tables at the L.E.D.A. Research Centre was instrumented with 34 sensors (92 channels), including Epson M-A352 QMEMS, Safran Colibrys VS1002, Analog Devices ADXL355, and PCB Piezotronics 356A17 accelerometers (Figure 7). The structure was subjected to progressively increasing shaking levels reproduced from the ground motion recorded during the 6 April 2009 Mw 6.3 L’Aquila earthquake, providing a benchmark for comparing different sensing technologies under both ambient-vibration and strong-motion conditions.

Although all sensors adequately reproduced the structural response during strong-motion shake-table tests, significant differences emerged under the low-amplitude vibration conditions relevant to Operational Modal Analysis. Consistent with their lower overall noise levels, the M-A352 QMEMS and PCB Piezotronics 356A17 sensing systems provided the clearest stabilization diagrams and the most robust identification of the principal modal frequencies. In contrast, the VS1002 and, particularly, the ADXL355 exhibited a reduced capability to resolve weak structural modes, highlighting the critical role of self-noise in OMA and SHM applications.

As illustrated in Figure 7, these observations are fully consistent with the self-noise characteristics discussed in Section 2 and support self-noise densities below approximately 1 μg/√Hz—and preferably below 0.5 μg/√Hz—as practical performance targets for reliable modal identification and long-term tracking of structural dynamic properties.

### 3.2. Timing Synchronization Performance

Accurate timing synchronization is a key requirement for seismic monitoring, Operational Modal Analysis (OMA), and distributed Structural Health Monitoring (SHM) systems. In particular, applications involving phase measurements, transfer-function estimation, modal identification, and array processing demand a high degree of phase coherence and, in many cases, sub-millisecond synchronization accuracy [40,41,42,43,44]. Table 3 summarizes indicative synchronization accuracies for representative applications in seismic monitoring, OMA, and SHM, together with their typical frequency ranges of interest. The reported values should be regarded as indicative engineering guidelines rather than strict synchronization requirements. They were established by considering qualitative and quantitative indications from published studies on modal identification, distributed sensing networks, and seismic monitoring systems [20,28,29,40,41,42,43,44], together with the experimental results obtained in the present study. However, the actual timing accuracy required for a specific application depends on the frequency range of interest and on the sensitivity of the target parameters to phase and synchronization errors [40].

To assess the timing performance of the proposed Smart Sensor Box, a series of laboratory and field experiments was carried out under both GNSS-disciplined and GNSS-denied operating conditions. The investigations included direct oscilloscope measurements of the 1PPS signals, characterization of the holdover behavior, and waveform-based analyses including phase estimation, coherence analysis, and sub-sample cross-correlation techniques.

The current Smart Sensor Box architecture employs a conventional voltage-controlled crystal oscillator (VCXO) as the local timing reference during GNSS holdover operation. Accordingly, Section 3.2.1 and Section 3.2.2 present the experimental characterization of the current VCXO-based timing solution, whereas Section 3.2.3 presents the laboratory evaluation of a temperature-compensated VCXO (TCVCXO) selected for the next hardware revision of the Smart Sensor Box.

#### 3.2.1. GNSS-Disciplined Operation

Under standard operating conditions, synchronization among sensing nodes is maintained through the GNSS-disciplined Time Base Unit described in Section 2.2. The timing subsystem was first characterized by means of laboratory measurements performed independently of the sensing chain. Direct oscilloscope measurements of the PPS signals revealed a relative timing difference of only 54 ns between two independent GNSS receivers, while the timing offset between the GNSS-derived PPS and the synthetic PPS generated by the acquisition unit was approximately 14.2 μs.

**Figure 8:**
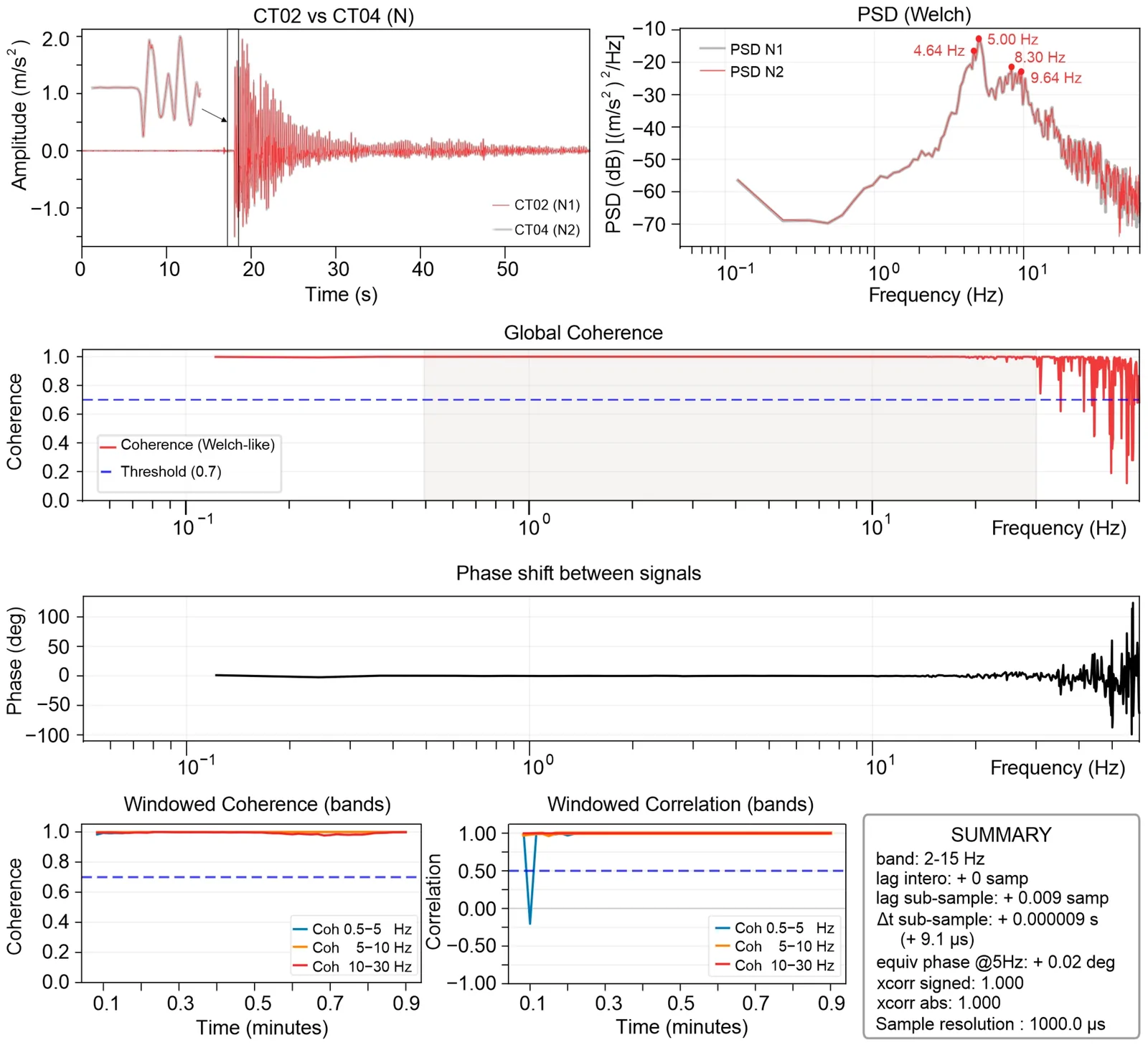
Example of synchronization validation between two co-located Smart Sensor Boxes (CT02 and CT04) operating under GNSS-disciplined timing and sampling simultaneously at 1000 Hz. The upper panels compare the recorded N–S acceleration waveforms and their PSDs, showing agreement in both the time and frequency domains. In the upper-left panel, the arrow indicates the enlarged view of the signal onset, while the vertical black lines delimit the time interval shown in the inset. The two central panels report the global coherence and phase difference between the two signals, with coherence values close to unity and negligible phase shifts across the frequency band of interest. The lower panels show the evolution of coherence and cross-correlation within selected frequency bands (0.5–5 Hz, 5–10 Hz, and 10–30 Hz), confirming stable synchronization throughout the recording. Sub-sample cross-correlation analysis yields an estimated residual relative timing offset of 0.009 samples (approximately 9.1 μs), with a correlation coefficient of 1.0 and an equivalent phase difference of approximately 0.02° at 5 Hz.

Synchronization performance was also assessed through coherence and cross-correlation analyses of simultaneously recorded vibration signals acquired from the N–S components of two co-located Smart Sensor Boxes. A dedicated processing workflow combining Welch coherence estimation, phase analysis, and sub-sample cross-correlation interpolation was developed to estimate relative timing offsets with a resolution significantly finer than the sampling interval. Synchronization tests performed on simultaneously operating GNSS-disciplined nodes revealed residual relative timing offsets of only a few microseconds, typically below 10 μs (Figure 8). At a sampling frequency of 1000 Hz, this corresponds to approximately 0.01 sample, confirming sub-sample temporal alignment between acquisition units.

The results indicate very high coherence and close agreement between the two independent estimation techniques. Similar behavior was observed for different components and frequency bands. Experiments were performed at both 200 and 1000 Hz sampling frequencies. At 200 Hz, relative delays estimated through sub-sample techniques generally remained within a few tens of microseconds and exhibited high temporal stability.

Comparable results were obtained at 1000 Hz, although the higher sampling frequency made the phase and cross-correlation estimates more sensitive to the selected bandwidth and signal characteristics. As illustrated in Figure 8, coherence values close to unity and very small phase-derived lags indicate that the proposed timing architecture provides synchronization accuracy compatible with distributed seismic monitoring, array processing, OMA, and SHM applications.

#### 3.2.2. GNSS-Denied Conditions

For dynamic identification measurements associated with SHM, sensors are frequently deployed inside buildings, underground environments, and cultural heritage structures where continuous GNSS reception cannot always be guaranteed. In these scenarios, maintaining synchronization stability for several hours after the temporary loss of the external timing reference becomes a key requirement, particularly for OMA, phase measurements, and transfer-function estimation.

**Figure 9:**
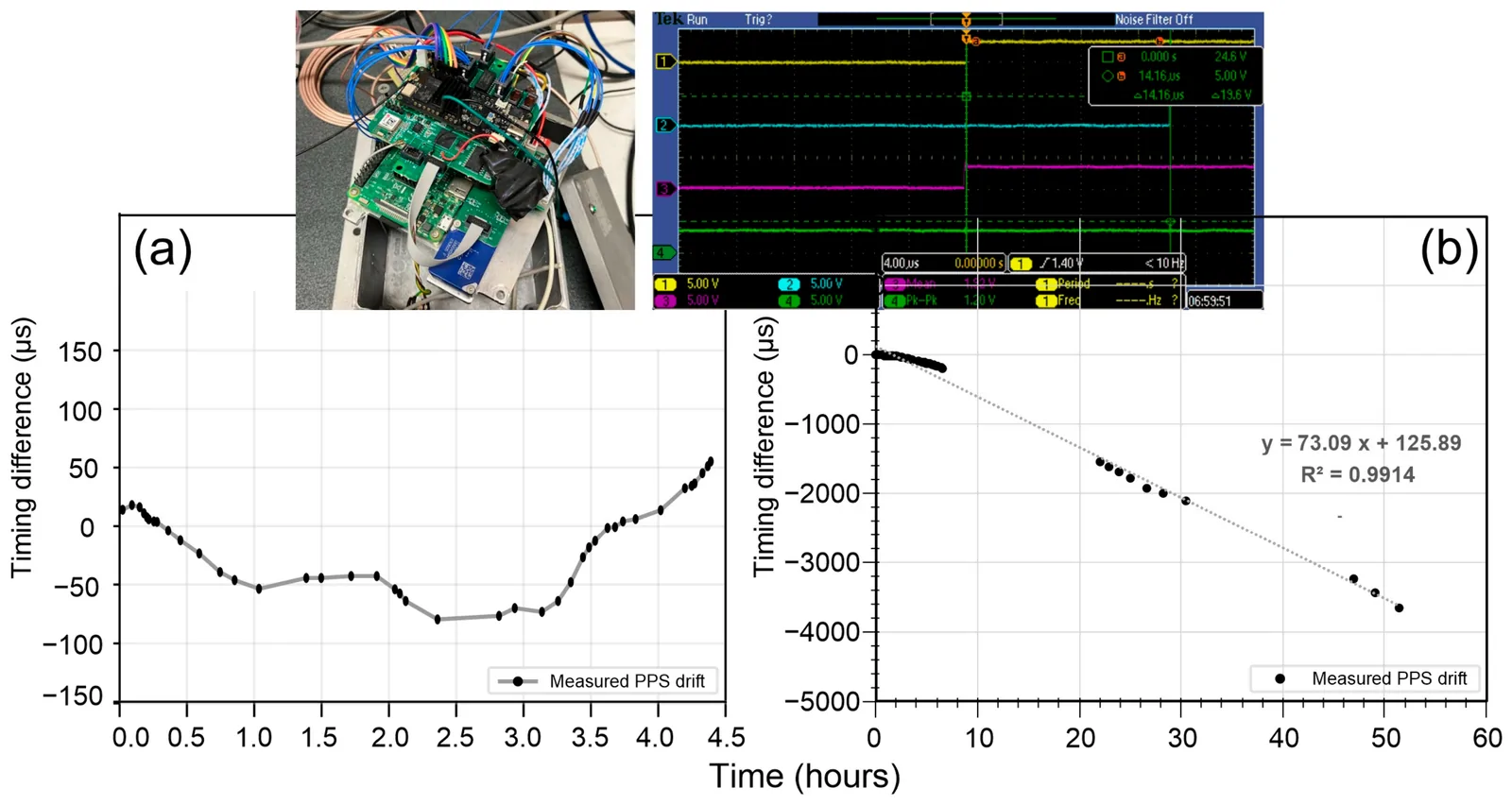
Comparison of the holdover performance of the standard VCXO and the TCVCXO characterized in Section 3.2.3. (**a**) Relative timing drift between the GNSS-derived PPS and the synthetic PPS generated by the VCXO-based timing unit during approximately 4.4 h of holdover operation following GNSS disconnection. (**b**) Timing drift of the TCVCXO during more than 50 h of autonomous operation following GNSS disconnection. In both experiments, timing drift was determined from direct oscilloscope measurements of the temporal separation between the rising edges of the GNSS-derived and synthetic PPS signals. The dashed line in panel (**b**) indicates the linear regression of the measured TCVCXO timing drift (R^2^ = 0.991).

The intrinsic holdover performance of the VCXO was first characterized through laboratory measurements (Figure 9a).

Following GNSS disconnection, timing stability was primarily governed by the frequency stability of the local oscillator and its temperature dependence. During the approximately 4.4 h holdover experiment, the relative timing error remained within ±80 μs, demonstrating good short-term stability (Figure 9a and Table 4). These laboratory measurements characterize the intrinsic holdover behavior of the VCXO-based timing unit relative to the GNSS-derived PPS and provide a reference for interpreting the subsequent long-term experiment between two co-located Smart Sensor Boxes.

**Figure 10:**
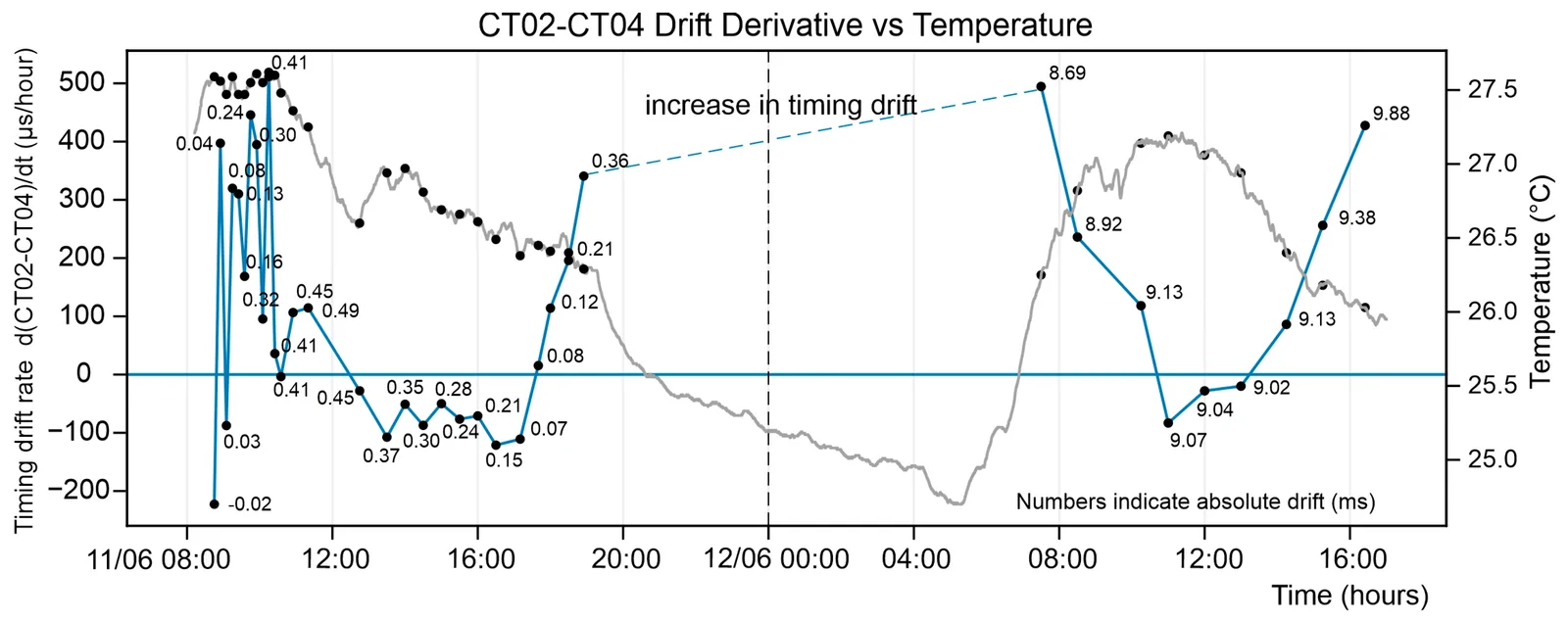
Drift rate of the CT02–CT04 timing offset during GNSS holdover operation and comparison with the ambient temperature measured by the BME280 sensor. The left axis represents the rate of change in the CT02–CT04 timing offset, *d*(CT02–CT04)/*dt*, expressed in μs h^−1^; positive values indicate an increasing timing offset, whereas negative values indicate a decreasing offset. The right axis shows the corresponding ambient temperature evolution. Numerical labels at selected measurement epochs indicate the absolute timing drift (ms) and should therefore be distinguished from the instantaneous drift rate represented on the left axis.

To investigate this aspect, a series of measurements was carried out using a pair of co-located Smart Sensor Boxes under controlled GNSS-denied conditions, complementing the laboratory tests described above. Two identical acquisition units (CT02 and CT04) were initially synchronized through GNSS. Subsequently, the GNSS receiver of one unit (CT04) was disconnected, while the second unit (CT02) remained continuously GNSS-disciplined and served as the timing reference. Relative timing offsets between the two acquisition units were then estimated over more than 32 h using phase analysis and sub-sample cross-correlation techniques applied to transient signals and ambient vibrations. The results summarized in Figure 10 and Table 4 document the evolution of the relative timing offset during extended holdover operation.

Following GNSS disconnection, the relative timing offset between CT02 and CT04 remained limited to a few hundred microseconds during the first hours of operation, increasing from approximately 160 μs after 1 h to about 300–450 μs after 2–3 h. The temporal evolution of the delay was generally gradual but non-monotonic, without abrupt discontinuities or cycle slips, indicating that the observed drift was primarily controlled by the intrinsic stability of the local oscillator and its sensitivity to temperature variations. Such timing offsets remain compatible with many modal-identification applications, although their influence on phase-sensitive distributed analyses depends on the frequency range and required synchronization accuracy. Figure 10 provides additional insight into the holdover behavior by comparing the drift rate of the CT02–CT04 timing offset with the ambient temperature measured by the Bosch BME280 sensor. Although the cumulative timing drift increased from less than 0.5 ms on 11 June to nearly 10 ms on 12 June, the corresponding drift rate remained within the same order of magnitude, generally below ±500 μs h^−1^. This observation suggests that the oscillator frequency offset remained relatively stable during prolonged holdover operation, while the absolute timing error progressively accumulated over time. The comparison with ambient temperature further indicates that thermal variations contribute to the observed drift evolution, although the relationship appears nonlinear and partially hysteretic.

For typical temporary OMA surveys lasting approximately 2–3 h, the accumulated timing offset remains within the synchronization accuracy generally considered suitable for modal identification and SHM applications, providing sufficient temporal stability for distributed measurements without continuous GNSS visibility at each sensing node. Over longer periods, however, the cumulative timing error progressively increases, reaching approximately 9 ms after about 24 h of autonomous operation (Figure 10 and Table 4), consistent with the expected behavior of a standard VCXO operating in holdover mode. While the measured performance is fully compatible with temporary indoor monitoring campaigns lasting several hours after an initial GNSS synchronization phase, it becomes less suitable for long-duration distributed OMA and SHM measurements under prolonged GNSS-denied conditions.

The current Smart Sensor Box architecture, based on a standard VCXO characterized by a frequency stability of about ±50 ppm, already provides adequate timing stability for the intended seismic monitoring, OMA, and short-term SHM applications. However, to further improve holdover performance during prolonged GNSS outages, a high-stability temperature-compensated VCXO (TCVCXO) was experimentally evaluated.

#### 3.2.3. Comparison of VCXO and TCVCXO Holdover Performance

While the standard VCXO-based architecture exhibited high synchronization accuracy under GNSS-disciplined operation, its holdover performance can be considered adequate only for limited-duration applications, including temporary seismic monitoring campaigns typically lasting approximately 1–2 h and short-term OMA and SHM measurements. The accumulated timing drift and apparent temperature sensitivity observed during prolonged GNSS-denied operation therefore motivated the experimental evaluation of a higher-stability temperature-compensated VCXO (TCVCXO) for the next hardware revision of the Smart Sensor Box. A commercially available TCVCXO operating at 30.720 MHz and providing a nominal frequency stability of approximately ±0.05 ppm over the 0–55 °C range was evaluated. Its voltage-controlled tuning range is compatible with the existing GNSS-disciplined timing-control architecture. By comparison, the non-temperature-compensated VCXO currently adopted in the Smart Sensor Box is specified for an overall frequency stability of ±50 ppm over its standard 0–70 °C operating range. After verifying the TCVCXO functional compatibility as a replacement for the standard VCXO within the existing timing architecture, the laboratory investigation focused on its intrinsic holdover performance. Since only a single TCVCXO prototype was available for testing during this study, synchronization measurements between two independent acquisition units could not be performed. Following initial GNSS synchronization, GNSS disciplining was disabled, and the timing unit was operated in holdover mode, while the drift of the synthetic PPS relative to the GNSS-derived PPS reference was continuously measured using an oscilloscope. Figure 9b summarizes the timing drift measured during more than 50 h of autonomous operation.

In contrast to the non-monotonic drift observed for the standard VCXO (Figure 9a), the TCVCXO exhibited a smooth and predominantly monotonic timing evolution without abrupt discontinuities or cycle slips, indicating stable and predictable free-running operation. The accumulated timing offsets were approximately 22 μs after 2 h, 51 μs after 3 h, 194 μs after 6 h, and 1.7 ms after 24 h (Figure 9b and Table 4). Relative to the initial synchronization, these values correspond to cumulative average drift rates increasing from approximately 0.003 μs s^−1^ (11 μs h^−1^) after 2 h to 0.009 μs s^−1^ (32 μs h^−1^) after 6 h and approximately 0.020 μs s^−1^ (71 μs h^−1^) after 24 h. Despite the lower drift rates observed during the initial hours, the timing-offset evolution over the complete measurement interval was well approximated by a linear trend (R^2^ = 0.991), consistent with a small residual frequency offset.

During the first 6 h, the measured offset remained equivalent to less than 0.2 samples at sampling frequencies between 200 and 1000 Hz and was compatible with the synchronization performance typically adopted for distributed OMA and SHM measurements. After 24 h, the accumulated offset remained substantially lower than that observed for the standard VCXO-based architecture during extended holdover operation.

Under the ambient laboratory conditions of the experiment, the timing evolution showed no evident short-term perturbations suggestive of a strong temperature influence. However, because the TCVCXO temperature was not monitored, the thermal contribution to the residual offset cannot be determined from the available measurements. Under GNSS-disciplined operation, temperature-induced frequency variations are continuously corrected by the timing-control loop, thereby minimizing their effect on synchronization. During GNSS-denied holdover, by contrast, timing stability depends primarily on the intrinsic performance of the oscillator. Moreover, controlled cyclic high–low-temperature tests have not yet been performed at the complete Smart Sensor Box level. Consequently, the present investigation does not fully characterize temperature-dependent timing drift, thermal hysteresis, heating–cooling asymmetry, or cycle-to-cycle repeatability, which are recognized phenomena in quartz oscillators [45,46].

The preliminary measurements demonstrate substantially improved TCVCXO holdover performance and support its adoption in the next revision of the Smart Sensor Box timing architecture. Once a second TCVCXO-based Smart Sensor Box becomes available, two nominally identical units will be subjected simultaneously to repeated high–low-temperature cycles in a climatic chamber. One unit will operate under continuous GNSS-disciplined conditions, whereas the other will operate in GNSS-denied holdover following initial GNSS synchronization. Their timing offsets will be continuously measured against an independent GNSS reference. These tests will quantify the thermal effects at the complete system level and distinguish the corrective effect of GNSS disciplining from the intrinsic temperature-dependent holdover behavior of the TCVCXO-based architecture.

Overall, replacing the standard VCXO with the TCVCXO can substantially improve timing stability during temporary GNSS interruptions while maintaining performance compatible with short-term modal-identification, phase-based, and distributed seismic and SHM measurements. The improved holdover stability may also enable less frequent GNSS resynchronization or duty-cycled GNSS operation, potentially reducing power consumption without compromising the required timing performance.

#### 3.2.4. First-Order Assessment of Timing-Induced Phase Errors in Modal Identification

To complement the representation of timing errors as fractions of a sampling interval in Table 4, a first-order analytical relationship was used to evaluate their potential effect on the phase information employed in modal identification. Following the time-shift formulations presented in [47,48], a constant timing offset Δt_i_ affecting the i-th acquisition channel can be represented in the frequency domain as

(1) $${\tilde{X}}_{i}\;(f)=X_{i}\;(f)\;e^{-j2\pi f\Delta ti}$$

where *Xi* (*f*) and ${\tilde{X}}_{i}$ (*f*) denote the synchronized and time-shifted channel responses, respectively. The corresponding phase error is

Δ*ϕ_i_* (*f*) = 2*π f* Δ*t_i_* (2)

or, in degrees,

Δ*ϕ_i_* ^(*deg*)^ (*f*) = 360 *f* Δ*t_i_* (3)

For distributed measurements, the relevant quantity is the relative timing offset between channels,

Δ*t_ij_* = Δ*t_i_* − Δ*t_j_* (4)

which produces an inter-channel phase error

Δ*ϕ_ij_* (*f*) = 2*πf*Δ*t_ij_* (5)

This relationship is consistent with the time-lag formulation adopted in [42]. A common timing shift affecting all channels equally does not modify their relative phase relationships. A constant relative timing offset does not alter the amplitude spectra or the locations of the spectral peaks of the individual channels and is therefore not expected, by itself, to significantly bias natural-frequency estimates. However, it modifies the relative phases between channels and may consequently affect cross-spectral matrices and complex mode-shape estimates [42,47,48].

Using Equation (5), the relative timing offset of approximately 10 μs measured under GNSS-disciplined operation corresponds to phase errors of approximately 0.004°, 0.018°, and 0.036° at illustrative modal frequencies of 1, 5, and 10 Hz, respectively. In the conservative worst-case scenario in which two units exhibit opposite timing deviations of ±10 μs relative to the GNSS reference, their relative offset may reach 20 μs, resulting in phase errors approximately twice those estimated for a 10 μs offset. These values are extremely small and are not expected to have an appreciable influence on natural-frequency or mode-shape estimates. If taken as representative of possible relative inter-channel offsets, the timing errors of 22–51 μs measured with the TCVCXO after 2–3 h of GNSS-denied holdover would correspond to phase errors of approximately 0.008–0.018° at 1 Hz, 0.040–0.092° at 5 Hz, and 0.079–0.184° at 10 Hz. For comparison, the relative offsets of 300–450 μs measured between the standard VCXO-based units after 2–3 h correspond to approximately 0.108–0.162°, 0.54–0.81°, and 1.08–1.62° at the same frequencies. After 24 h, the measured timing errors of approximately 1.7 ms for the TCVCXO and 9 ms for the standard VCXO would correspond to phase errors ranging from approximately 0.61° to 6.12° and from 3.24° to 32.4°, respectively, over the 1–10 Hz interval. Therefore, under GNSS-disciplined operation, the estimated inter-channel phase errors are negligible across the illustrative 1–10 Hz modal-frequency range. In TCVCXO-based holdover mode, they remain very small over the first 2–3 h and are expected to have a negligible effect on natural-frequency estimates and only a very small influence on phase-sensitive mode-shape estimates in short-duration OMA and SHM measurements. Conversely, prolonged GNSS-denied operation may introduce increasingly significant phase errors, particularly at higher modal frequencies, although the estimated errors remain substantially smaller with the TCVCXO than with the standard VCXO. However, the propagation of timing errors into individual modal parameters, particularly mode shapes and damping ratios, is more complex and depends on the identification method, modal separation, signal-to-noise ratio, record duration, and temporal evolution of the clock error. A comprehensive application-specific analysis would require numerical simulations or reprocessing of experimental data with controlled timing perturbations; this lies beyond the scope of the present study and will be addressed in future work.

## 4. Field Applications and Emerging Monitoring Frameworks

### 4.1. Urban Seismic Observatories and Impact-Based Earthquake Early Warning

**Figure 11:**
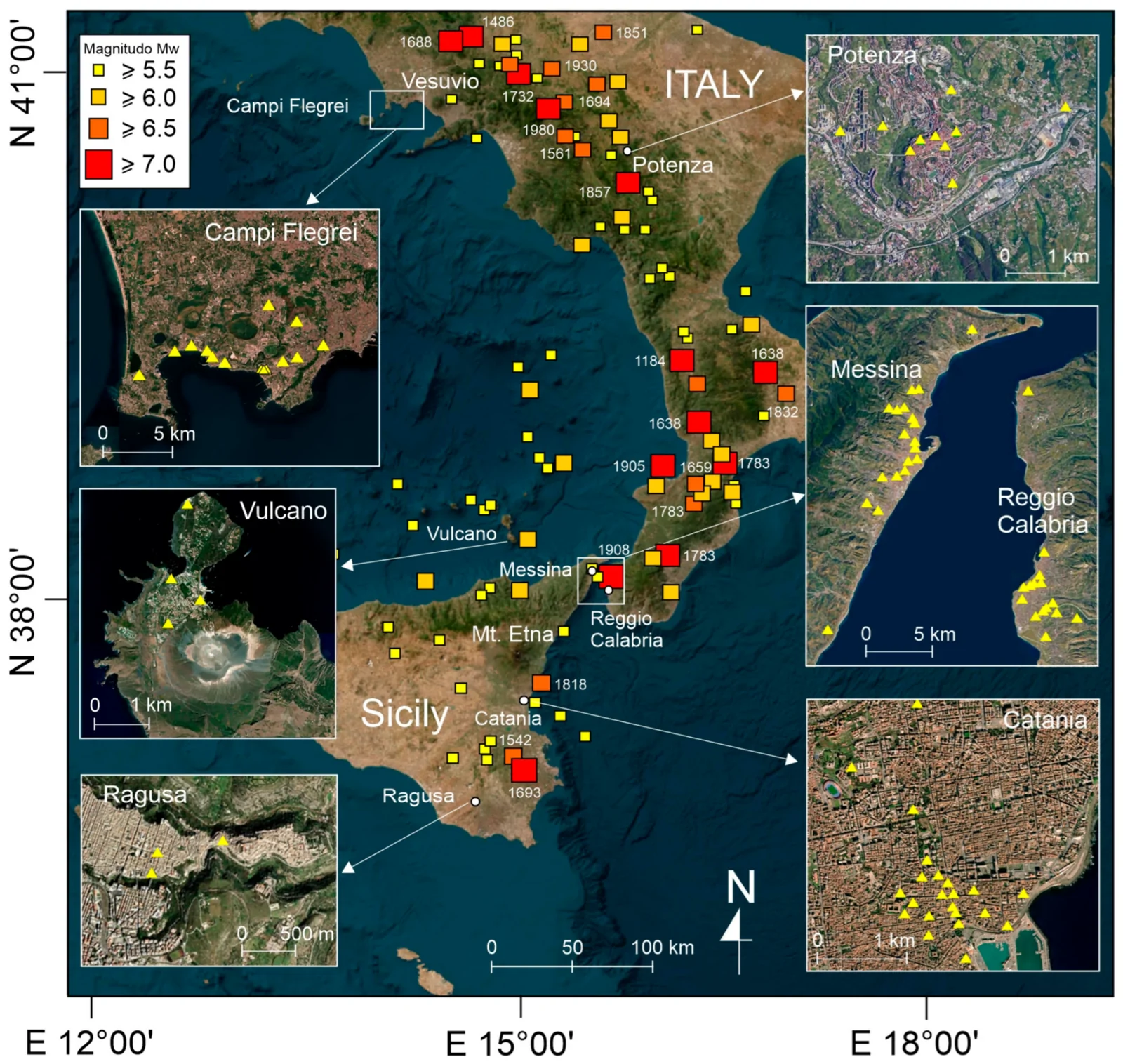
Southern Italy map with the spatial distribution of the urban seismic observatories of CMSU. Colored squares indicate the epicenters of historical earthquakes with magnitude larger than 5.5. In the insets are shown the investigated areas together with the station distribution (yellow triangles) within the urban environments of Catania, Campi Flegrei, Messina–Reggio Calabria, Potenza, the port of Vulcano, and Ragusa. In Catania, Potenza, and Ragusa, QMEMS sensors are integrated with force-balance and conventional MEMS accelerometers, whereas only QMEMS deployments are represented for the Messina–Reggio Calabria and Campi Flegrei volcanic area, where more than 50 QMEMS M-A352 accelerometers are currently in operation.

Recent advances in medium- to low-cost sensing technologies, together with improvements in communications, GNSS timing, edge computing, and data management, have made it possible to deploy dense urban networks capable of continuously monitoring earthquake ground motion within complex urban environments. Since 2022, southern Italy has seen the establishment of several urban seismic monitoring systems, starting with the Catania Urban Seismic Observatory (OSU-CT) [9]. These observatories provide high-resolution strong-motion datasets that not only improve the understanding of local site effects and the spatial variability of earthquake ground motion, but also enable new applications in Structural Health Monitoring (SHM) and impact-based Earthquake Early Warning (EEW). Figure 11 summarizes the geographical distribution of the realized Urban Seismic Observatories and monitoring networks.

Experience gained during the operation of the 22-station Catania Urban Seismic Observatory showed that the adoption of Epson M-A352 accelerometers, owing to their lower self-noise, consistently provided higher-quality seismic recordings and improved signal-to-noise ratios than other widely used low-cost MEMS accelerometers, including the Analog Devices ADXL355, which was also deployed throughout the network and, at several sites, in co-located installations. As a result, the M-A352 provides clearer phase arrivals and enables more reliable extraction of earthquake early-warning parameters over a broad range of earthquake magnitudes and source distances [9]. However, the observed signal quality ultimately depends on earthquake magnitude and depth, propagation effects, and local site conditions. Owing to the frequent microseismic activity of Mt. Etna, local earthquakes with magnitudes of approximately ML 2.0 are readily detected by the network, with clear P- and S-wave arrivals observed at epicentral distances of 5–10 km. Events of approximately ML 3.0 can also be reliably detected and characterized at distances of several tens of kilometers, enabling robust estimation of early-warning parameters from the first seconds of the recordings.

**Figure 12:**
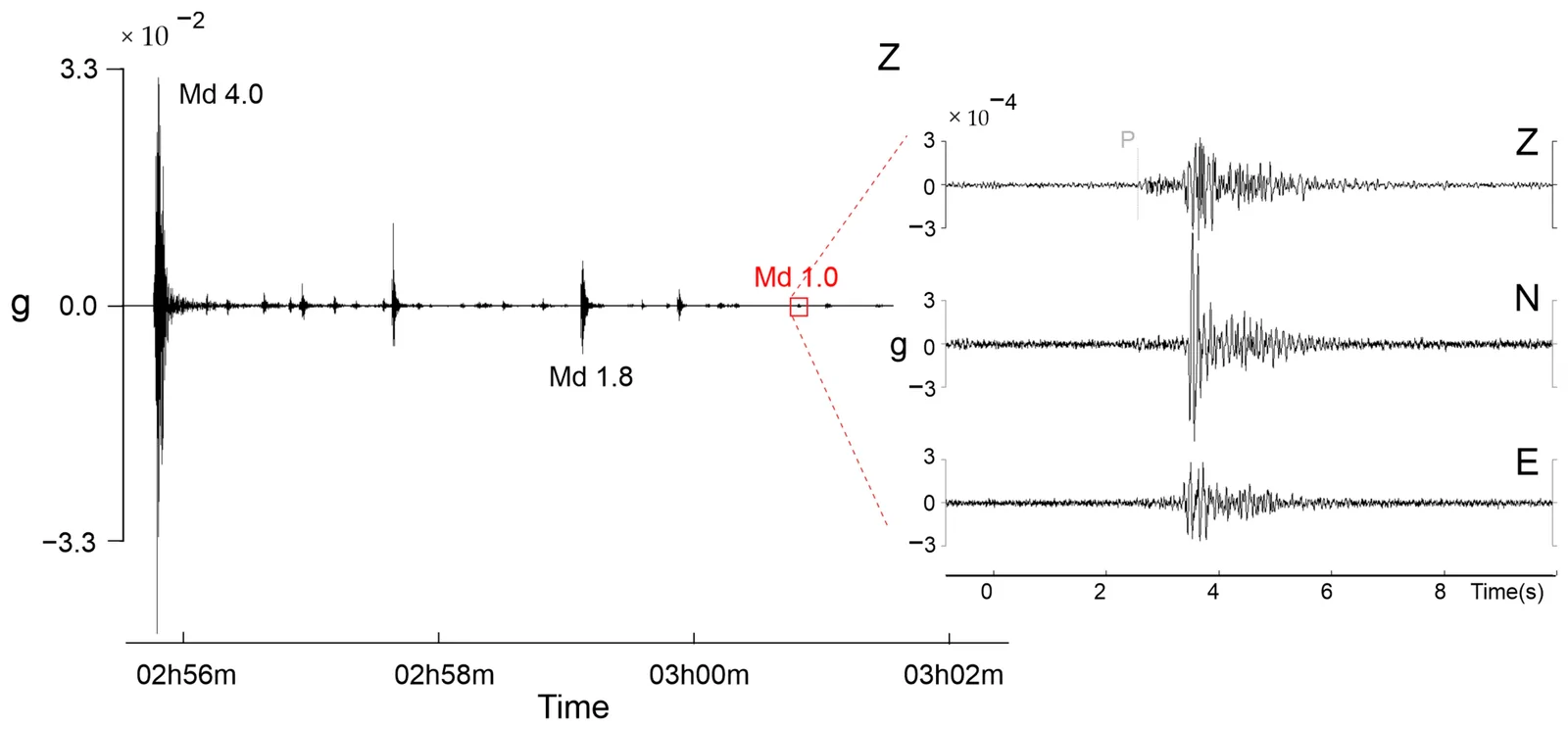
Example of continuous seismic recordings acquired at station OCF04 of the Campi Flegrei Urban Seismic Observatory using an Epson M-A352 QMEMS accelerometer. The left panel shows a continuous record collected on 1 September 2025, including the Md 4.0 earthquake together with several smaller local events. The panel on the right illustrates the three-component recording of a Md 1.0 earthquake, highlighting the capability of the QMEMS sensor to detect and accurately record very weak local seismic events.

Following the Catania deployment, the same QMEMS sensors have been employed in the other urban seismic observatories and in several SHM applications involving buildings, bridges, dams, and cultural heritage structures, further demonstrating their capability to accurately record both weak and strong motions. An example is provided by the Campi Flegrei Urban Seismic Observatory [37], established in 2024 in response to the ongoing bradyseismic unrest affecting the caldera. Owing to its predominantly shallow seismicity, with most earthquakes occurring at depths of less than 3 km, and the frequent occurrence of low-magnitude events, the Campi Flegrei area represents an ideal natural laboratory for assessing weak-motion monitoring capabilities. Continuous recordings acquired by the M-A352 QMEMS stations exhibit excellent signal-to-noise ratios, enabling the reliable detection and recording of local earthquakes with magnitudes around Md 1.0 and below (Figure 12).

The recorded waveforms exhibit a quality comparable to that of co-located broadband seismometers and force-balance accelerometers operated by INGV–Osservatorio Vesuviano and the Italian National Accelerometric Network (RAN), further validating the use of low-noise QMEMS technology for high-resolution seismic monitoring in complex urban volcanic environments with elevated ambient noise levels. Together with the permanent INGV-OV and RAN stations, the OCF network currently forms one of the densest urban strong-motion monitoring systems operating in Italy.

Following the successful deployment of the Catania and Campi Flegrei Urban Seismic Observatories, the most recent Messina–Reggio Calabria Urban Seismic Network represents the largest homogeneous deployment based on Epson M-A352 QMEMS accelerometers, comprising 38 stations predominantly installed in strategic buildings and schools. This dense urban network provides an ideal framework for investigating the spatial variability of earthquake ground motion at the city scale and complements conventional seismic microzonation studies. While seismic microzonation provides essential static information for territorial planning, Urban Seismic Observatories directly measure the actual spatial distribution of earthquake shaking within the built environment and provide the real-time information required for rapid impact assessment.

An example of these capabilities is provided by the automated analysis of recordings acquired by the Messina–Reggio Calabria Urban Seismic Network during the deep Mw 6.1 Tyrrhenian earthquake of June 2026, performed using an impact-based EEW platform named PSAVE, which is currently under evaluation. Despite similar hypocentral distances of approximately 270–280 km, the stations exhibited significant variability in recorded ground-motion amplitudes, highlighting the influence of local geological conditions and site effects. In particular, peak ground acceleration was dominated by the vertical component, consistent with the large focal depth of the event, whereas peak ground velocity showed a more balanced contribution from the three components and proved to be more representative of the ground shaking experienced across the urban area.

**Figure 13:**
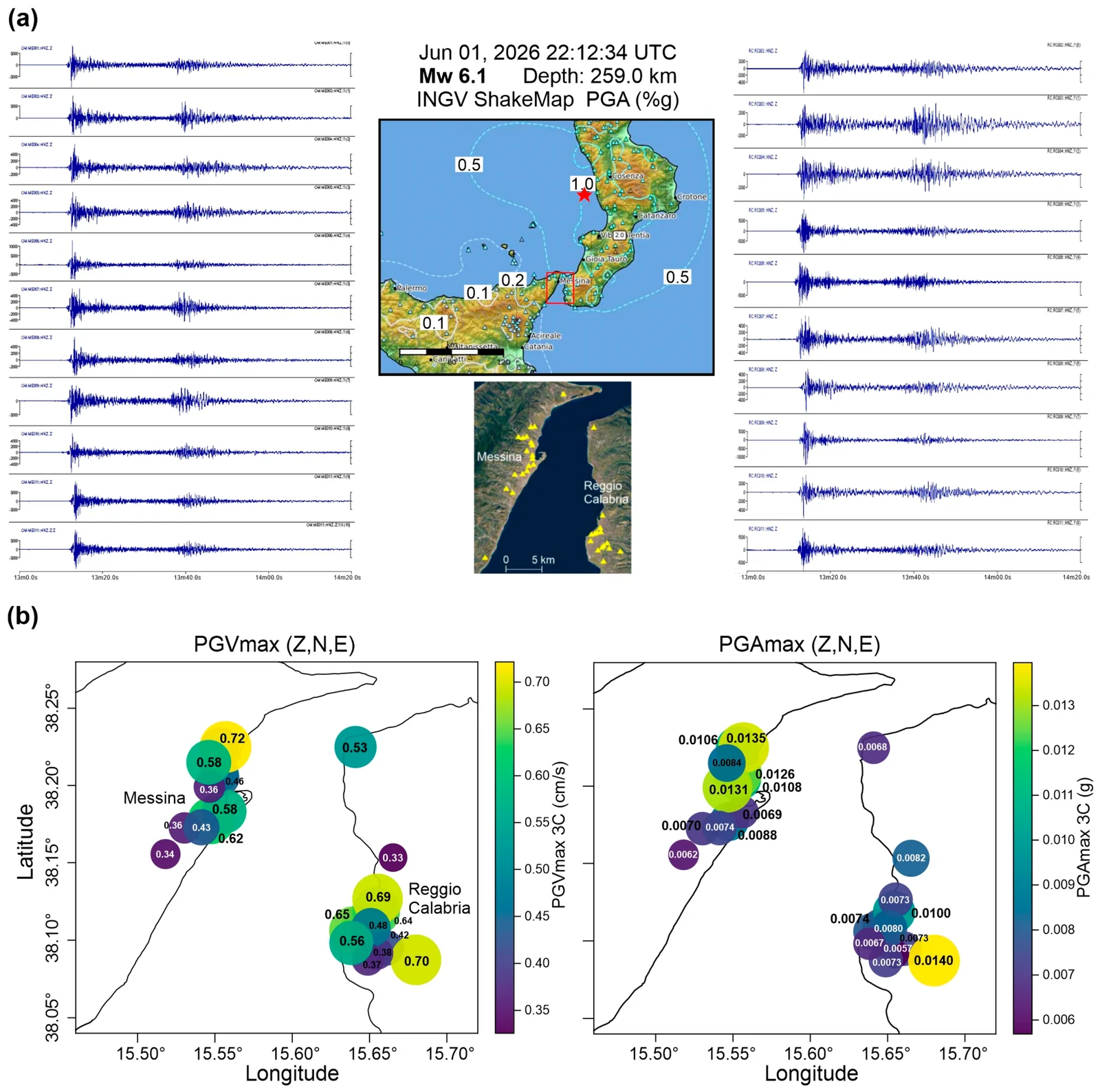
Response of the Messina–Reggio Calabria Urban Seismic Observatory (OSU) stations to the deep Mw 6.1 earthquake of 1 June 2026. (**a**) Overview of the event, showing the vertical (Z) component waveforms recorded at twenty OSU stations, the corresponding regional INGV ShakeMap (https://shakemap.ingv.it/data/46107472/current/products/pga.jpg; accessed on 18 July 2026), and the geographical distribution of the stations across the Messina Strait. Star indicates Epicenter. (**b**) Spatial distribution of the maximum PGV and PGA values. For each station, the reported values correspond to the maximum among the three components (Z, N, and E). Circle size and color are proportional to the measured amplitudes, while the numerical labels indicate the corresponding PGV (cm s^−1^) and PGA (% g) values.

Figure 13 summarizes the spatial distribution of the observed PGA and PGV values and illustrates the capability of the network to resolve local differences in ground motion over relatively short urban distances. These observations show how real-time dynamic recordings can directly capture local amplification effects and the spatial variability of earthquake shaking that may not be fully resolved by conventional microzonation studies. Consequently, Urban Seismic Observatories provide a valuable framework for understanding the interaction between earthquakes and the built environment, improving the seismic resilience of urban areas, and bridging the traditional gap between seismology and structural engineering.

These same Urban Seismic Observatories also provide a valuable testbed for the ongoing development and experimental validation of PSAVE. The platform builds upon the methodologies previously proposed in [49,50] while incorporating several methodological improvements. Unlike conventional source-based EEW approaches, the adopted strategy combines rapid source characterization with the direct estimation of expected shaking and impact parameters, providing information more closely related to the potential effects on buildings, infrastructure, and the built environment. To support prompt response, the first predictive EEW alerts are based on the initial P-wave signal issued at 1, 2, and 3 s after P-wave detection. These alerts provide progressively more reliable estimates of expected PGV, macroseismic intensity, and other early-warning parameters, with increasingly stable estimates generally obtained from 2 s onward, based on preliminary extensive tests performed in the Campi Flegrei area. Strong-motion parameters, including measured PGA and PGV, are subsequently evaluated from the incoming waveform and continuously updated as the event evolves. Depending on hypocentral distance and wave propagation, these parameters become progressively more representative of the actual ground shaking as additional waveform data are acquired. Further updates can be provided, for example, at 5 and 10 s, with strong-motion summaries progressively updated until the end of the event.

**Figure 14:**
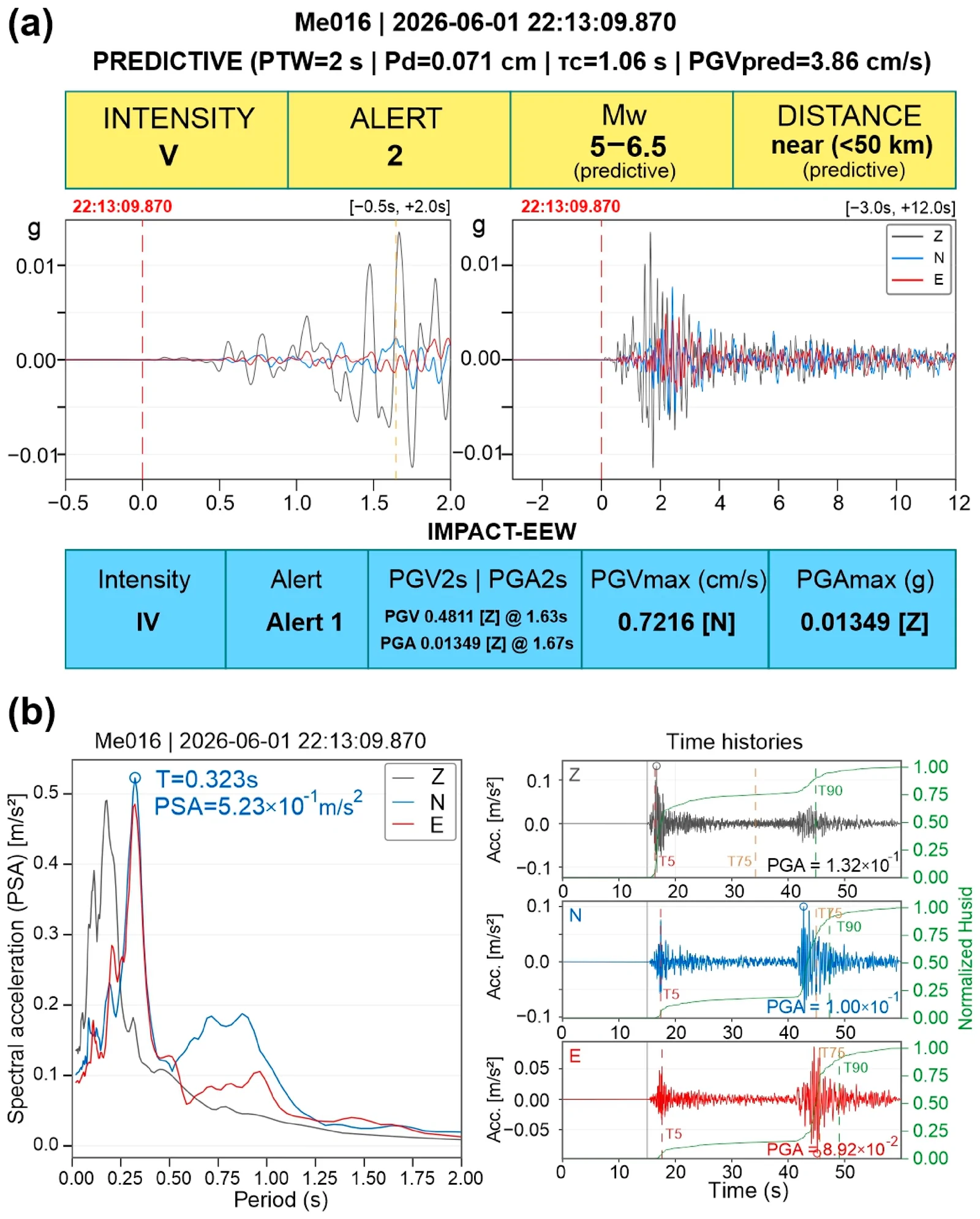
Example of the products generated by PSAVE at the ME016 station of the Messina network during the Mw 6.1 earthquake of 1 June 2026 (22:12:35 UTC), which occurred at a hypocentral depth of approximately 260 km. (**a**) PSAVE impact-based EEW summary showing both predictive and observed parameters; (**b**) pseudo-spectral acceleration curves and three-component acceleration time histories.

Figure 14 illustrates representative graphical products generated by PSAVE for station ME016 of the Messina Urban Seismic Observatory during the deep Mw 6.1 earthquake. For each station of the network, the system automatically provided real-time estimates of intensity, alert level, engineering-impact indicators, observed PGA and PGV values, pseudo-spectral acceleration curves, other engineering parameters, and three-component acceleration time histories. The figure demonstrates how the automatic system captures local variations in ground shaking and progressively transitions from predictive EEW estimates to direct impact characterization.

This approach represents a shift from conventional centralized architectures toward distributed intelligent sensing systems capable of performing local processing and decision support directly at the edge.

Through the combined use of the dual-core microcontroller and the embedded Single Board Computer, event detection, strong-motion parameter estimation, and preliminary impact assessment can also be performed locally, thereby reducing communication requirements and dependence on centralized processing. These capabilities facilitate the local implementation of PSAVE through the Smart Sensor Box architecture described in Section 2.2, which combines very low-noise QMEMS accelerometers, precise timing synchronization, and embedded processing capabilities.

In summary, the combination of dense Urban Seismic Observatories, low-noise QMEMS accelerometers, accurate timing synchronization, and embedded processing capabilities provides a scalable framework for strong-motion monitoring, rapid post-earthquake assessment, impact-based EEW, and future digital-twin applications for urban areas and critical infrastructures.

### 4.2. Structural Health Monitoring Applications

Building upon the experience gained from Urban Seismic Observatories, the same low-noise QMEMS technology and Smart Sensor Box architecture have progressively been extended to structural engineering applications involving ordinary buildings, critical infrastructures, and cultural heritage structures. Experimental and field observations consistently reveal that accelerometer self-noise is one of the primary factors governing sensor suitability for modal identification. Under ambient excitation conditions, structural responses may be only a few micro-g above the background noise level, making the instrumental noise floor particularly important for reliable identification and long-term tracking of modal properties. As discussed in Section 3.1, these effects were clearly observed during the dynamic testing campaign carried out on the 2:3 scale masonry building at the L.E.D.A. laboratory [39]. Subsequent comparisons between the M-A352 and the next-generation M-A370 highlighted the advantages associated with the lower self-noise of the new-generation sensor. These improvements were particularly evident for weak-vibration measurements performed on structures characterized by extremely low-amplitude responses and located in very quiet environments, where the recorded signals approached the instrumental noise floor of the M-A352 [38].

**Figure 15:**
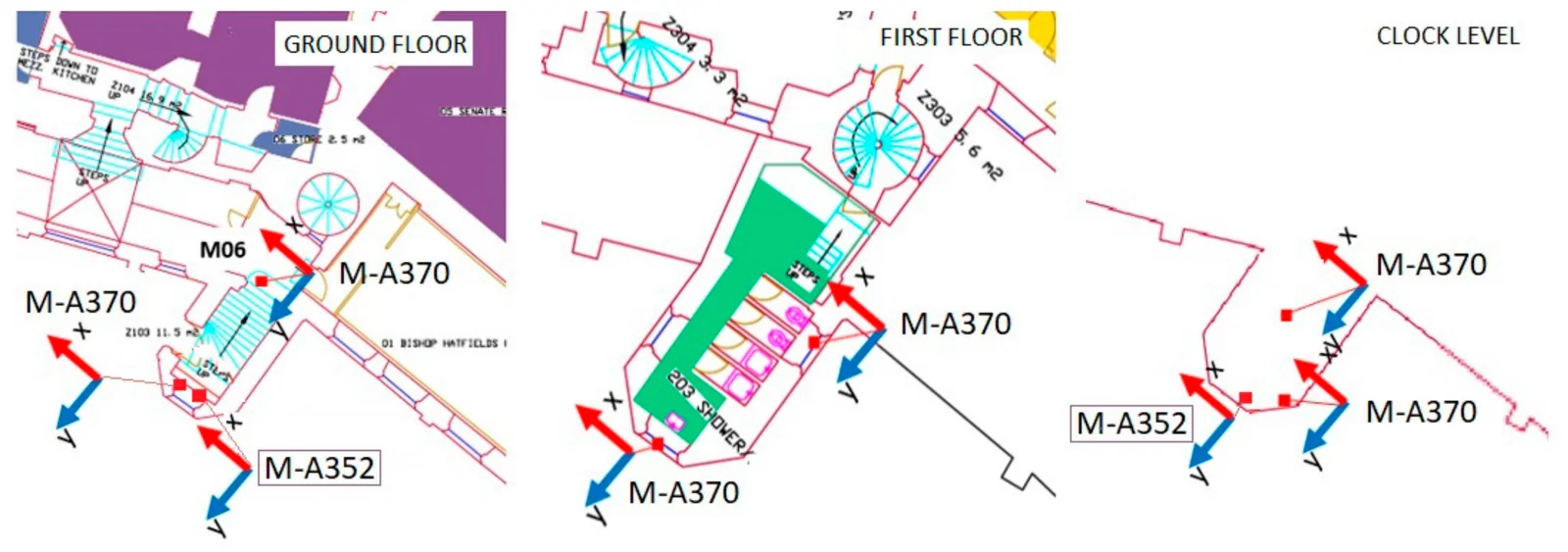
Adopted sensor layout at the clock tower during the ambient vibration campaign. M-A352 accelerometers were installed at the ground floor and at the clock level of the tower.

**Figure 16:**
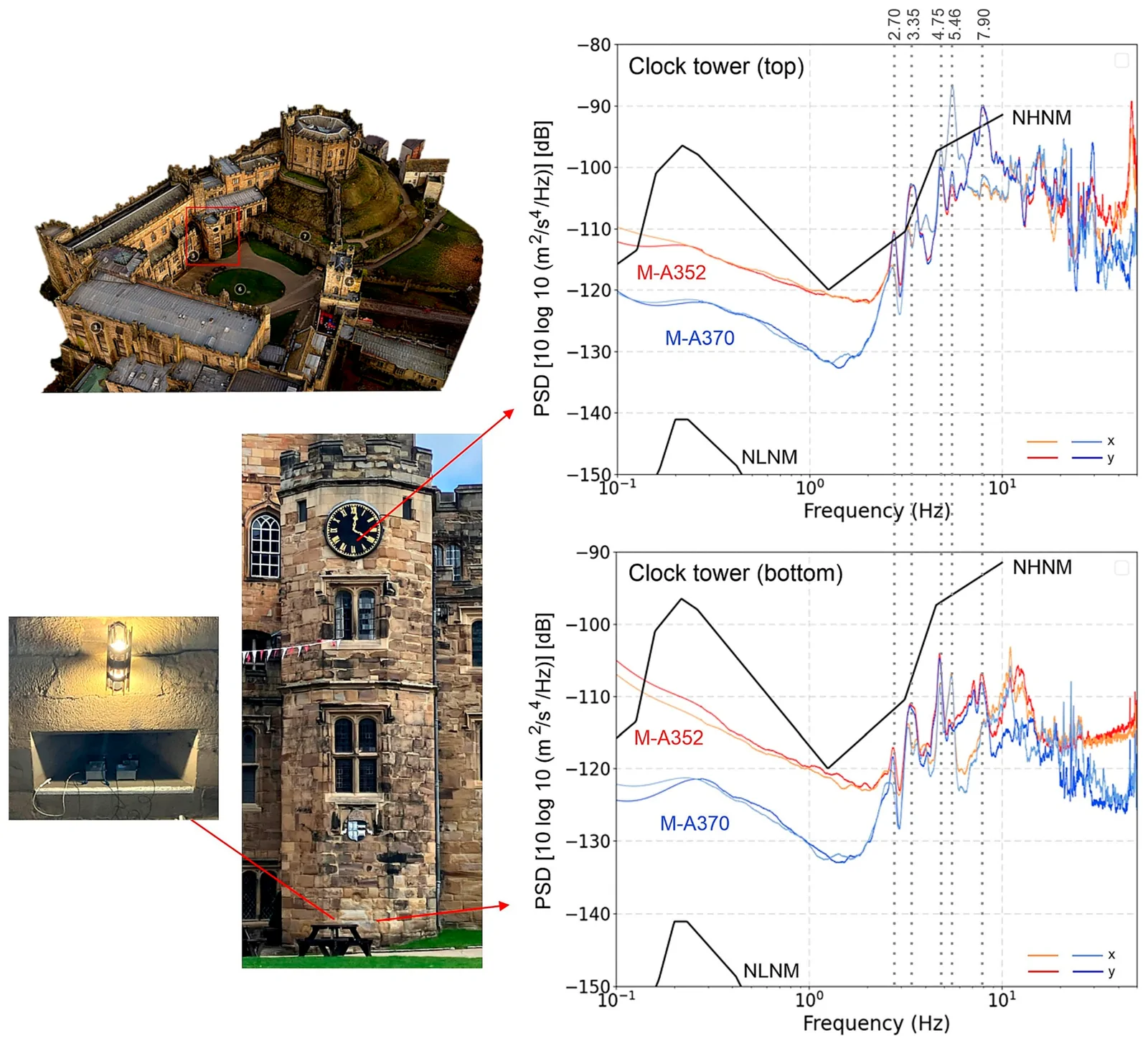
Experimental setup and comparison of the self-noise characteristics of the M-A352 and M-A370 accelerometers at the clock tower of Durham Castle (United Kingdom). Power spectral densities (PSDs) calculated from one hour (01:00–02:00 UTC) of ambient-noise recordings acquired simultaneously by co-located sensors installed at the top and base of the Clock Tower reveal several structural resonance frequencies between 2 and 10 Hz, with larger amplification observed at the top of the structure.

Additional supporting evidence was provided by the recent experimental campaign carried out at Durham Castle (United Kingdom), a UNESCO World Heritage masonry structure characterized by geometric complexity, very low vibration amplitudes, and the coexistence of global and local structural modes. During the campaign, a temporary network comprising ten stations equipped with next-generation M-A370 QMEMS accelerometers was deployed throughout the castle using multiple measurement layouts. This strategy enabled Operational Modal Analysis under ambient excitation conditions and the identification of both global and local vibration modes of the structure. Side-by-side measurements performed at the base and at the top of the clock tower (Figure 15) confirmed the benefits associated with the lower self-noise of the next-generation M-A370 (Figure 1 and Figure 16).

As shown in Figure 16, power spectral densities computed from one hour of ambient-noise recordings clearly reveal amplified structural resonances between approximately 2 and 10 Hz at the top of the tower. Under the extremely low-noise conditions encountered at the base of the structure, the M-A370 provides reliable measurements over a much broader frequency band and allows the observation of microseismic energy below 1 Hz, whereas the noise floor of the M-A352 limits the recognition of weak signals below approximately 1 Hz and above ~ 30 Hz. These results further confirm the potential of ultra-low-noise QMEMS technology for the dynamic characterization and long-term monitoring of complex heritage structures.

Another important application of QMEMS technology is represented by the UNESCO World Heritage Site of the Basilica of Saint Francis in Assisi, one of Italy’s most valuable cultural heritage monuments. Following the damage caused by the 1997 Umbria–Marche earthquake, a permanent monitoring system was progressively implemented to investigate the dynamic behavior of both the basilica and its bell tower [51].

The system integrates six triaxial Epson M-A552 accelerometers, an outdoor model incorporating the same QMEMS sensing element as the M-A352. The sensors are installed at different elevations of the bell tower together with the BAS01 seismic station located at its base. Continuous ambient-vibration monitoring enabled the identification of six stable vibration modes, mainly between 2 and 6 Hz, while long-term observations revealed seasonal variations primarily controlled by temperature effects, with no evidence of structural degradation [51]. Several earthquake recordings also revealed the progressive amplification of vibration amplitudes toward the upper levels of the bell tower, highlighting the capability of the system to characterize the dynamic response of the monument.

Beyond historical buildings and cultural heritage structures, the proposed sensing platform has also been successfully applied to the dynamic characterization and long-term monitoring of transportation infrastructures. A representative example is the reinforced-concrete arch bridge “Papa Giovanni XXIII” in Ragusa (southern Sicily, Italy), where the first prototype of the proposed Smart Sensor Box architecture was employed for both dynamic characterization and continuous Structural Health Monitoring (SHM) [52]. Before the installation of the permanent monitoring system, a campaign for dynamic characterization with the OMA was conducted under different environmental and traffic conditions to identify the principal dynamic characteristics of the bridge and to optimize the sensor layout for the permanent monitoring network [52].

**Figure 17:**
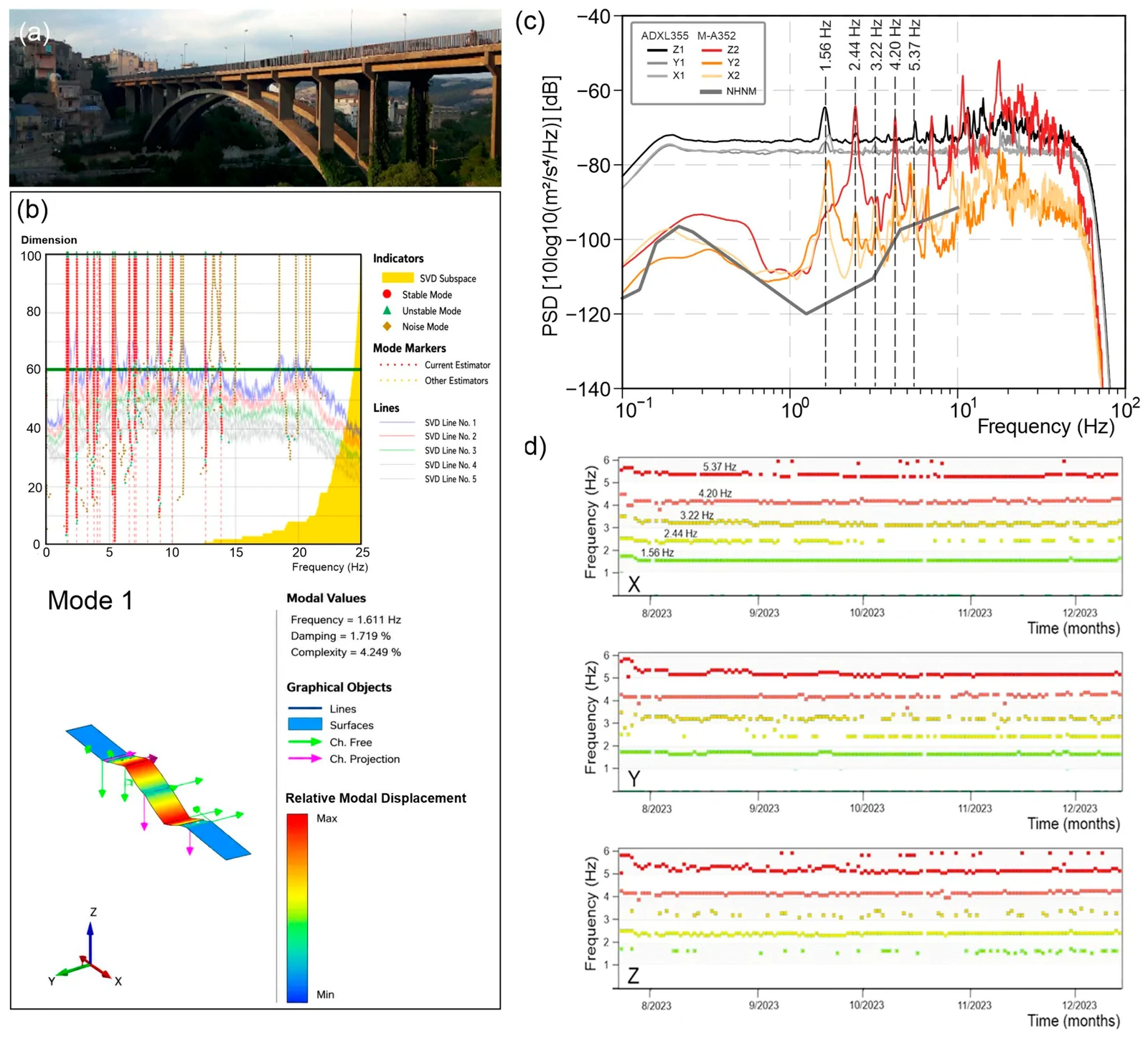
Structural Health Monitoring of the “Papa Giovanni XXIII” reinforced-concrete arch bridge in Ragusa (southern Italy). (**a**) General view of the bridge. (**b**) Stabilization diagram obtained from Operational Modal Analysis during the dynamic characterization together with the corresponding first bending mode shape identified at 1.61 Hz. (**c**) Comparison of power spectral densities computed from one hour of nighttime ambient-vibration recordings acquired by co-located ADXL355 and M-A352 accelerometers, highlighting the lower self-noise and improved detectability of structural resonances provided by the M-A352 sensor. (**d**) Automatic tracking of the first five modal frequencies over several months for the three components at a representative monitoring node.

Figure 17a,b show the bridge together with the corresponding modal identification results obtained during this preliminary investigation. Following the dynamic characterization, a permanent monitoring system based primarily on eleven low-noise M-A352 accelerometers was deployed on the structure, enabling continuous vibration measurements and automatic tracking of the structural dynamic properties over time. For comparative purposes, ADXL355 accelerometers were also installed alongside the M-A352 sensors. Figure 17c compares the power spectral densities computed from one hour of nighttime ambient-vibration recordings acquired at a representative monitoring node using co-located ADXL355 and M-A352 accelerometers. Several structural resonances clearly emerge in the M-A352 spectra, whereas they are partially or completely masked by the higher noise floor of the ADXL355. Automatic tracking of the identified modal frequencies over several months (Figure 17d) further confirmed the remarkable stability of the bridge dynamic properties and highlighted the suitability of low-noise QMEMS accelerometers for OMA and continuous SHM [52].

**Figure 18:**
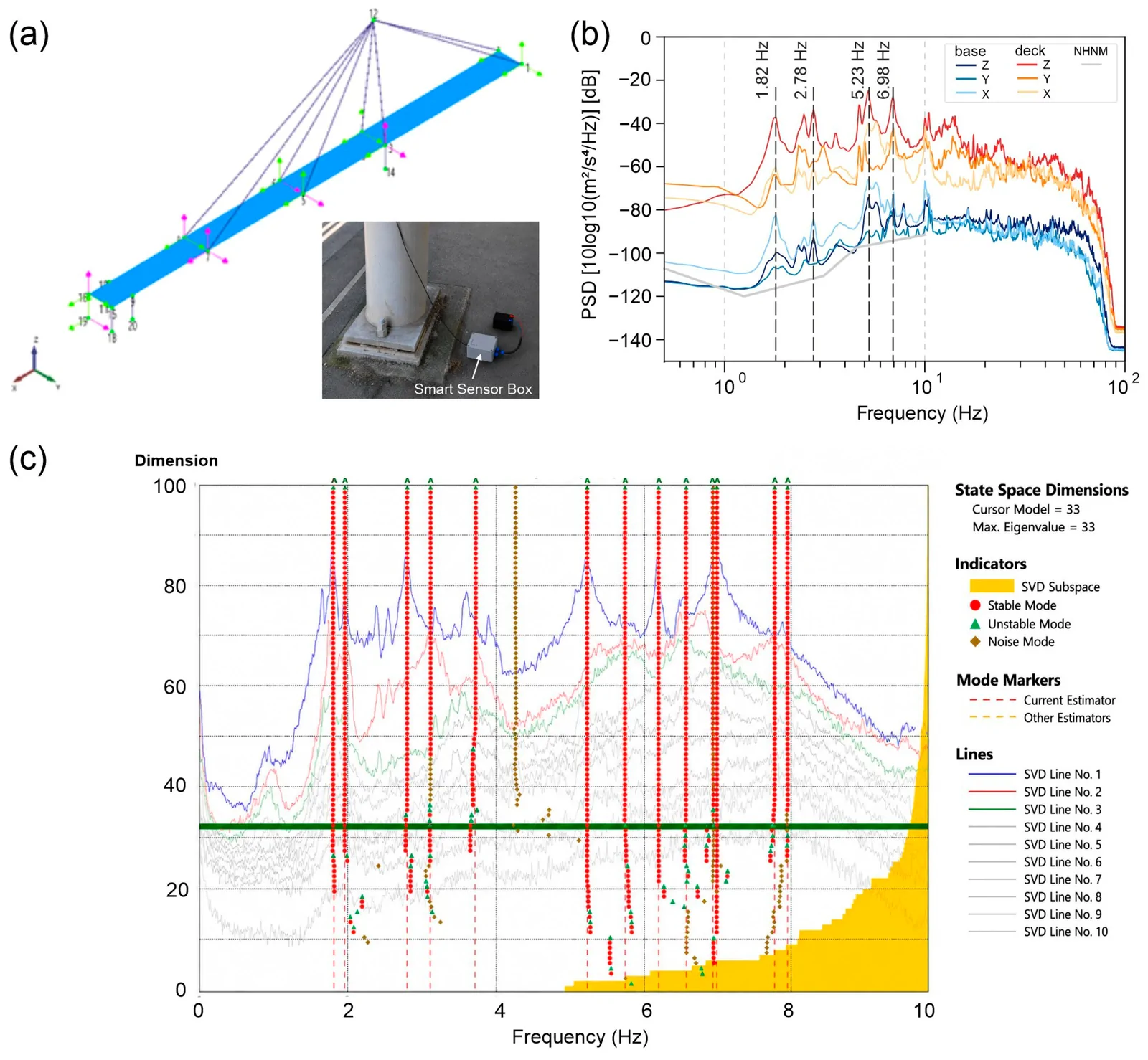
(**a**) Schematic representation of the cable-stayed steel pedestrian bridge in the United Kingdom used as an additional field validation case study of the proposed Epson M-A370 QMEMS sensing platform. The arrows indicate the orientation of the measurement axes at each location where a Smart Sensor Box was installed. The inset photograph shows the Smart Sensor Box (gray box) and the battery deployed at the base of the bridge. (**b**) Power spectral densities (PSDs) measured on the bridge deck and at the ground reference station, highlighting the amplification of the bridge response within the principal modal frequency range. (**c**) Stabilization diagram obtained using the Stochastic Subspace Identification Unweighted Principal Components (SSI-UPC) method. Stable physical poles are highlighted by red markers, confirming the robust identification of the bridge modal frequencies under ambient vibration conditions.

An additional field experiment using the Epson M-A370 QMEMS accelerometers was recently conducted on 16 July 2026 on a cable-stayed steel pedestrian bridge in the UK. Ambient vibration measurements were performed to characterize the dynamic behavior of the bridge through OMA, providing an additional independent validation of the proposed sensing platform for SHM applications. The bridge is approximately 60 m long and 3.4 m wide. Its structural system consists of a steel deck supported by six stay cables connected to an asymmetric pylon. Owing to access restrictions, sensors could not be installed on the pylon; therefore, the measurement layout focused on the bridge deck, where nine Smart Sensor Boxes were deployed together with one reference station installed on the adjacent ground, providing a total of 30 measurement channels (Figure 18a).

The measurement layout was designed considering the structural configuration of the bridge, its expected modal behavior, and site-access limitations. Particular attention was devoted to the spatial distribution of the sensors to maximize the observability of the principal deck vibration modes, while accounting for the inability to instrument the pylon.

This measurement strategy is consistent with the general monitoring framework discussed in the Introduction, where sensor placement represents a key component of reliable OMA and SHM. The bridge was monitored for approximately three hours under normal operating conditions, without interrupting pedestrian traffic or requiring interaction with bridge users. The Smart Sensor Box architecture performs local data acquisition at each measurement point, eliminating the need for cabled connections between sensors and a centralized data acquisition system. The power spectral densities shown in Figure 18b indicate that the bridge deck exhibits an amplification of approximately 40 dB relative to the ground reference station within the principal modal frequency range.

The PSD measured at the ground reference station also reveals a weaker structural response, reflecting the dynamic coupling between the bridge and its supports. The stabilization diagram obtained using the Stochastic Subspace Identification Unweighted Principal Components (SSI-UPC) method (Figure 18c) indicates excellent identification quality. Stable physical poles appear already at relatively low model orders and remain consistent up to the highest model orders considered, indicating high-quality measurements obtained under ambient excitation.

**Figure 19:**
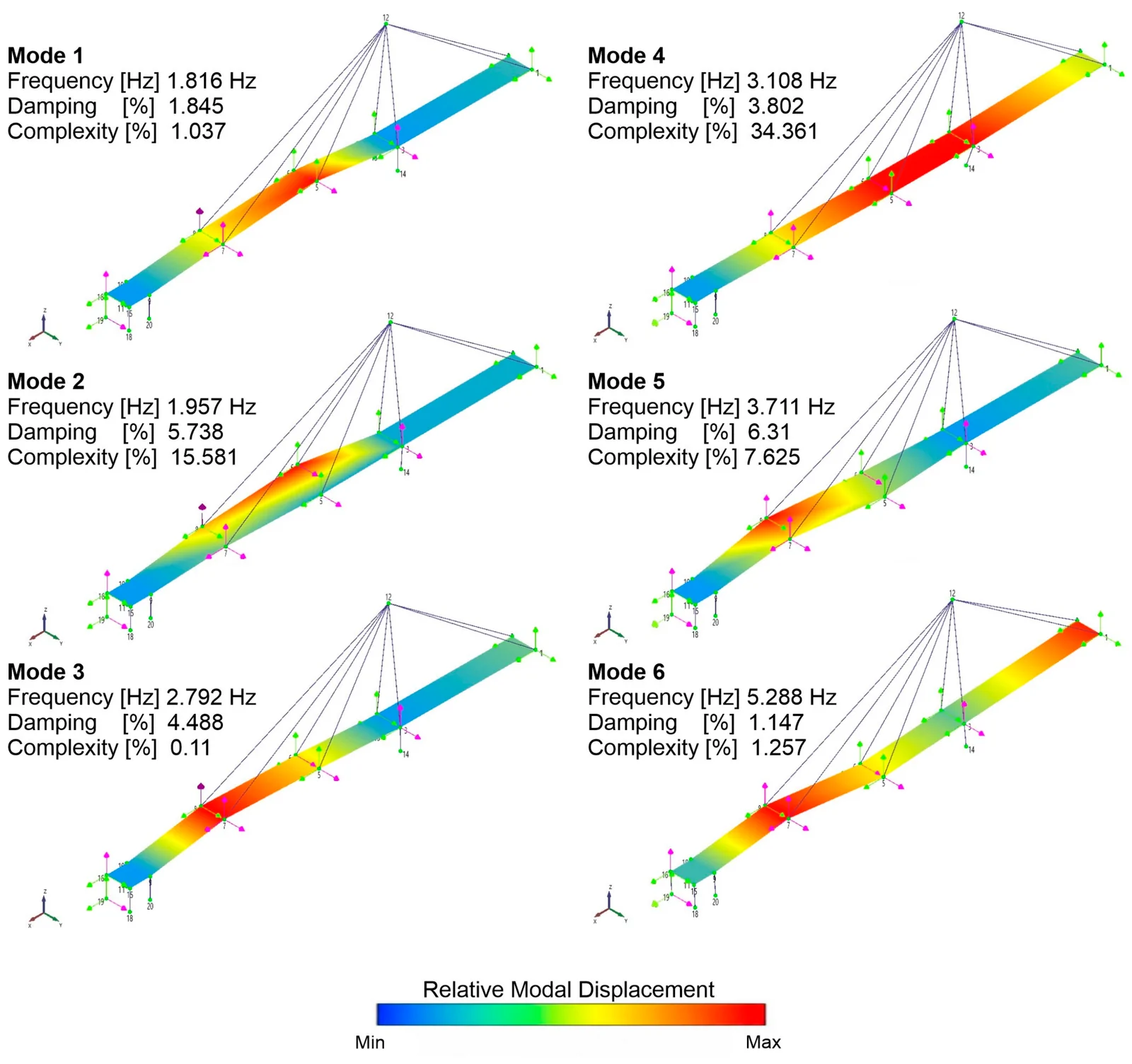
First six identified mode shapes of the pedestrian bridge obtained by means of the SSI-UPC method. The corresponding natural frequencies, damping ratios, and modal complexity values are reported for each identified mode.

Twelve stable modes were identified within the 0–8 Hz frequency band, clustered into two distinct, closely spaced frequency clusters—roughly 1.816–3.711 Hz and 5.228–7.770 Hz—rather than a single evenly spaced sequence. Figure 19 presents the first six identified mode shapes together with their corresponding natural frequencies, damping ratios, and modal complexity values. This clustering is consistent with what is generally reported in the literature for cable-stayed decks suspended from multiple stays, where each stay anchorage effectively behaves as an elastic support. The first and third mode shapes further suggest that the deck does not behave as a continuous beam suspended by multiple stays, but rather as a sequence of elastically supported spans.

Overall, these results provide further independent validation of the proposed QMEMS-based sensing platform, confirming its capability to perform reliable OMA under ambient excitation, even on lightweight civil engineering structures characterized by closely spaced vibration modes. While the previous examples support the application of QMEMS technology to the monitoring of individual structures, the same sensing platform can also be deployed at the urban scale, where seismological monitoring and Structural Health Monitoring (SHM) converge within a common observational framework.

One of the most significant applications of the proposed sensing platform is in the Campi Flegrei caldera, where the current bradyseismic unrest and increasing shallow seismicity have motivated the development of an integrated seismic and structural monitoring system [37].

Since 2024, some residential and strategic buildings have been progressively instrumented with M-A352 accelerometers within the framework of the Campi Flegrei Urban Seismic Observatory, combining dense accelerometric measurements with vibration-based SHM techniques. Particular attention has been devoted to representative reinforced-concrete buildings in the Bagnoli district, where “simplified” monitoring systems employing a limited number of sensors have been installed at the foundation level and at the upper floors. Although this approach provides only an approximate description of the structural response, it significantly reduces installation costs and enables the deployment of distributed monitoring systems on an urban scale. Continuous recordings acquired under ambient vibrations and during local earthquakes allow the identification and tracking of modal frequencies and provide information on the evolution of structural dynamic properties.

**Figure 20:**
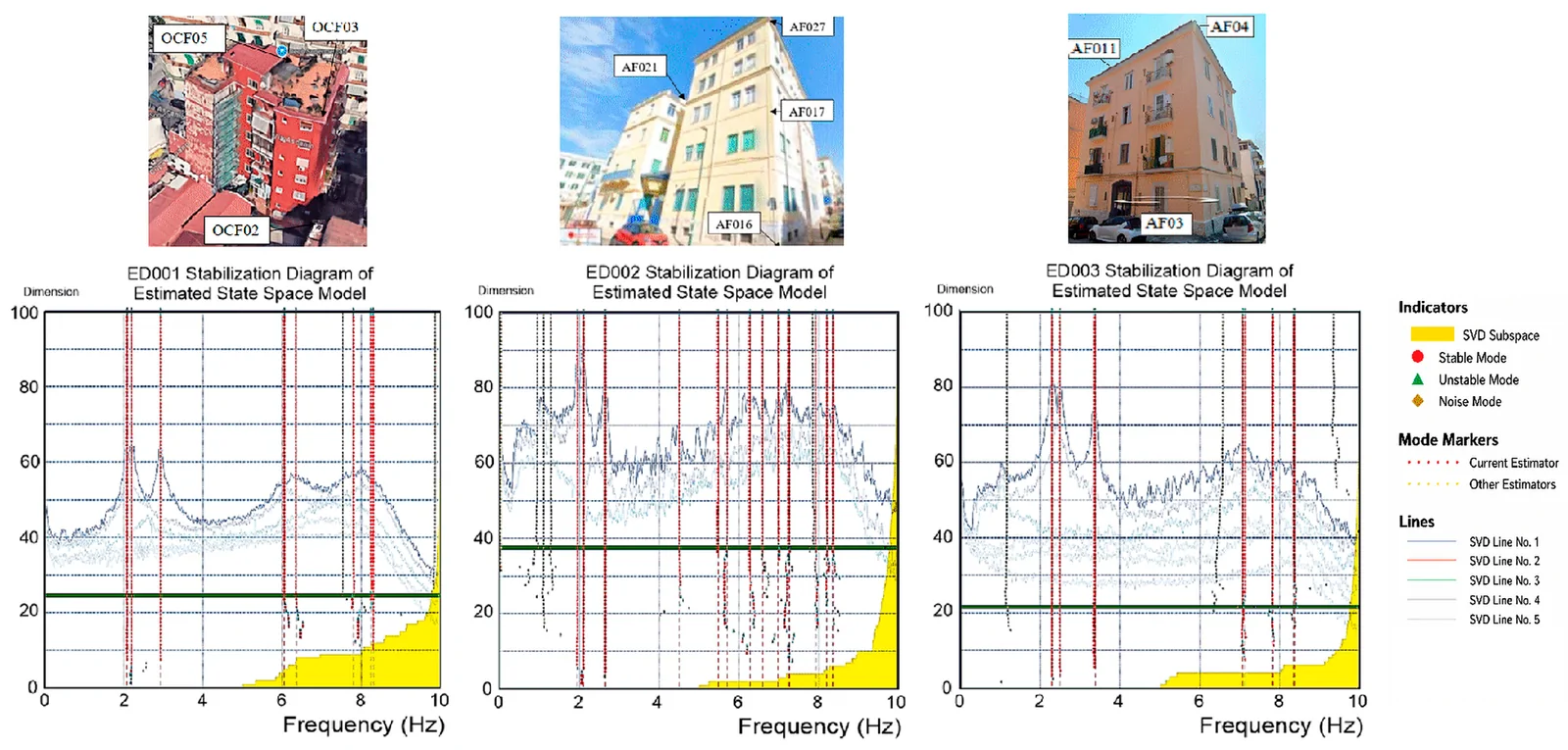
Instrumented buildings ED001–ED003 and corresponding stabilization diagrams obtained from ambient vibration measurements through the SSI-UPC method. Across the three buildings, the stable poles identify modal frequencies ranging from 1.9 to 3.4 Hz, corresponding to two flexural modes and one torsional mode for each building (see Table 5).

Figure 20 illustrates three representative monitored buildings (ED001–ED003) together with the corresponding stabilization diagrams obtained from ambient vibration measurements using the SSI-UPC method. In all cases, stable poles clearly reveal two predominant flexural modes and a torsional mode, highlighting the capability of the proposed QMEMS-based monitoring system to identify the dynamic properties of ordinary reinforced-concrete buildings under operational conditions. The modal parameters identified for the three buildings are summarized in Table 5. Fundamental frequencies range between approximately 1.9 and 3.4 Hz, while damping ratios are generally close to 1–2%. The complexity indices remain generally very small, confirming the reliability of the identified modes and the predominance of classical vibration behavior.

The frequent occurrence of shallow earthquakes during the ongoing bradyseismic unrest offers the opportunity to investigate not only ambient vibrations but also the structural response under real seismic excitations. Owing to the large number of events recorded since 2020, the integration of the Urban Seismic Observatory with local SHM installations enabled the investigation of the relationship between ground motion and structural demand [37].

In particular, acceleration-spectrum-based parameters exhibited stronger correlations with average inter-story drift than conventional peak values such as PGA, suggesting that spectrum-based intensity measures may provide more representative indicators of structural demand and suitable proxies for simplified vulnerability assessment and rapid post-earthquake evaluation.

**Figure 21:**
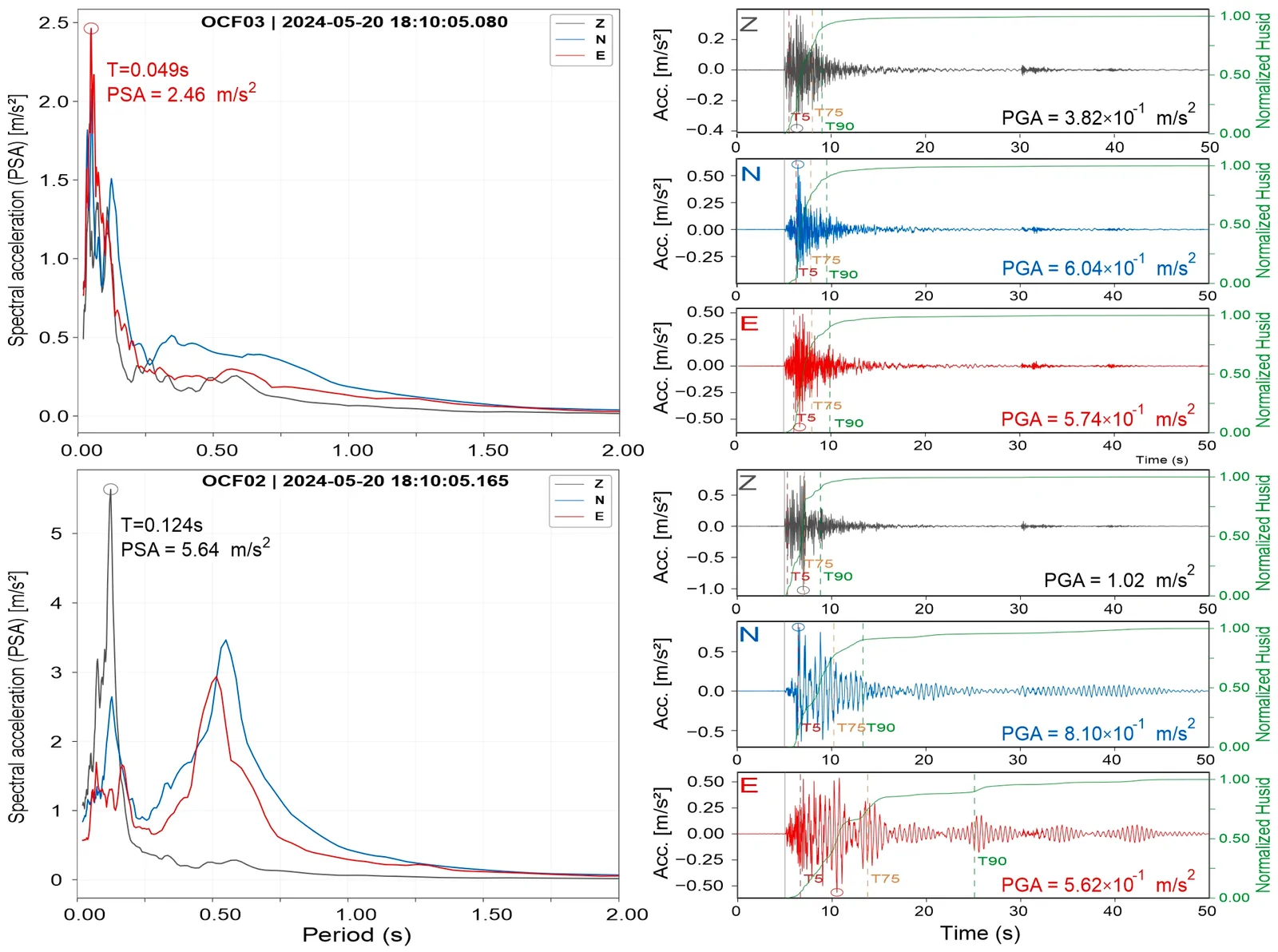
Pseudo-acceleration response spectra and three-component acceleration time histories of the Md 4.4 earthquake of 20 May 2024 recorded at stations OCF03 (top panels, basement level) and OCF02 (bottom panels, rooftop level) installed in building ED001 (see Figure 20). The Husid plot (green line), representing the cumulative energy release of the signal, is superimposed on the time histories and was used to estimate the significant durations T5, T75 and T90. Comparison between the basement and rooftop recordings highlights the amplification effects associated with the dynamic response of the structure.

Real earthquake recordings also provided the opportunity to investigate the structural response under actual seismic loading. Recordings acquired in building ED001 during the Md 4.4 earthquake of 20 May 2024 revealed clear amplifications between basement (OCF03) and rooftop (OCF02) stations and highlighted additional spectral peaks associated with the dynamic behavior of the structure (Figure 21).

**Figure 22:**
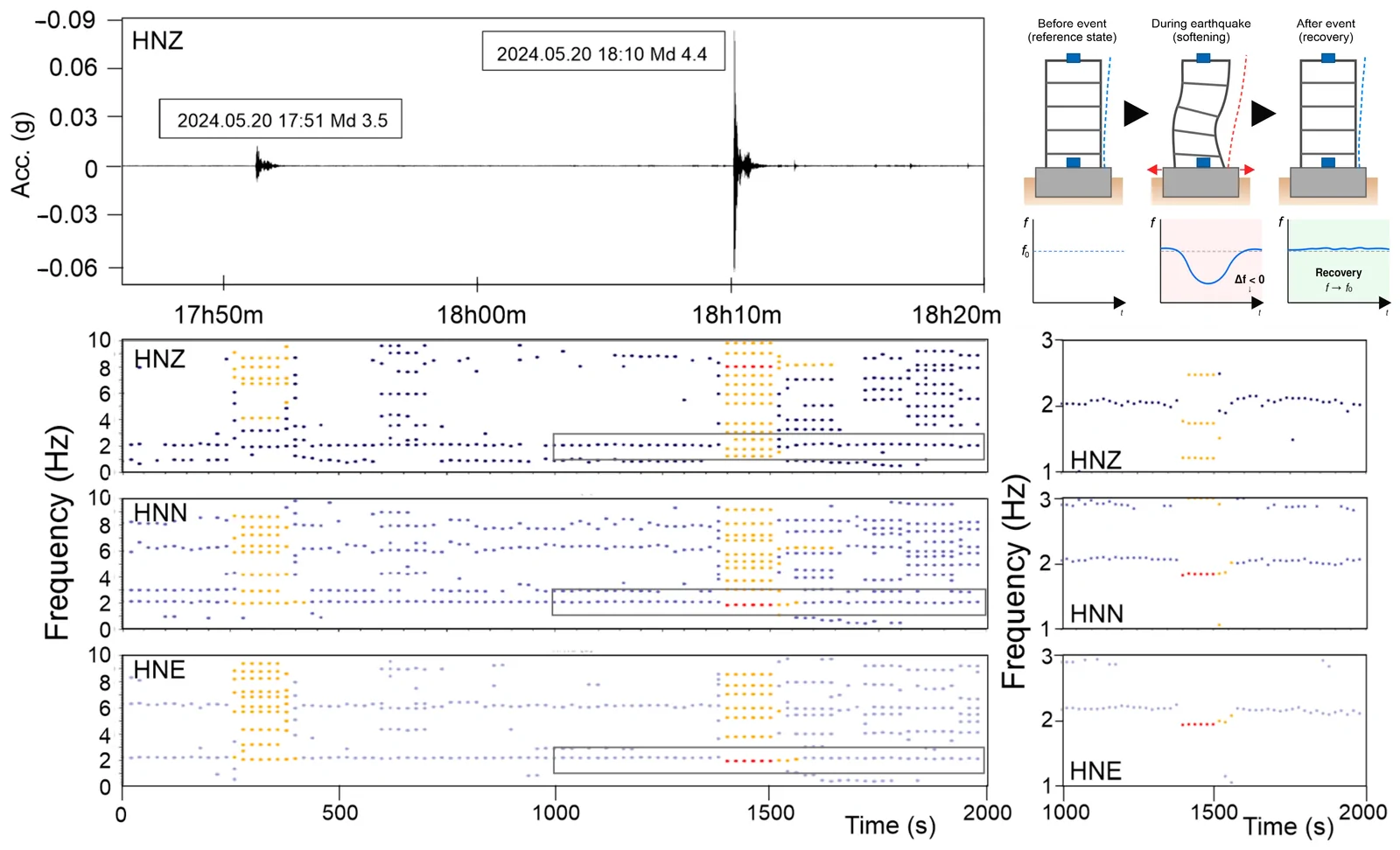
Automatic tracking of the modal frequencies of building ED001 derived from PSD analysis during the Md 3.5 and Md 4.4 earthquakes of 20 May 2024. The upper panel shows the vertical acceleration record (HNZ), while the lower panels display the evolution of the spectral peaks (dots) identified on the three components (HNZ, HNN, and HNE). The orange and red dots indicate two automatic alert levels associated with magnitude thresholds of Md ≥ 3.0 and Md ≥ 4.0, respectively. Zoomed views (lower-right panels) highlight the temporary decrease in the first modal frequency during the Md 4.4 earthquake and its subsequent recovery. Zoomed views (right panels) highlight the temporary decrease in the first modal frequency during the Md 4.4 earthquake and its subsequent recovery. The conceptual sketch in the upper-right corner illustrates the interpreted structural behavior before, during, and after the earthquake. During strong shaking, the temporary reduction in structural stiffness, potentially associated with soil–structure interaction and nonlinear structural response, produces a decrease in modal frequency; its subsequent return toward the pre-event value indicates recovery of the original dynamic properties and no evidence of permanent damage.

Continuous analysis of power spectral densities enabled the automatic tracking of modal frequencies over time. Figure 22 shows that the Md 4.4 earthquake produced a temporary shift in the first modal frequency, followed by a return to pre-event values. A similar but less pronounced behavior was also observed during the preceding Md 3.5 event.

Although the reduced sensor layout does not allow complete mode-shape identification, the information provided by the simplified monitoring configuration is sufficient to calibrate a simplified beam model and low-computational-cost fragility analyses [53]. While these approaches cannot replace detailed numerical simulations and conventional vulnerability assessments, they provide valuable information for rapid post-earthquake evaluations and large-scale applications. More generally, the Campi Flegrei installations represent one of the first operational examples in Italy of an integrated framework combining an Urban Seismic Observatory with distributed SHM, in which seismic ground motion and building response are investigated using the same technological infrastructure. Together with evidence obtained from recordings of actual earthquakes [54], these results highlight the potential of integrated seismic and structural monitoring to support resilience-oriented strategies and the development of simplified fragility models for urban-scale applications.

Overall, the applications discussed in this section indicate that accelerometer self-noise is a primary factor governing the reliability of vibration-based SHM. In general, self-noise densities below approximately 1 μg/√Hz, and preferably below 0.5 μg/√Hz, represent practical performance targets for robust identification and long-term tracking of modal properties under weak ambient excitation, whereas values approaching or below 0.1 μg/√Hz are desirable for the most demanding monitoring applications. Such performance enables the reliable observation of microtremors, microseismic signals, and low-amplitude structural resonances that might otherwise remain masked by instrumental noise. The experiences presented here demonstrate that low-noise QMEMS accelerometers, combined with precise timing synchronization and embedded processing capabilities, offer an effective framework for the monitoring of ordinary buildings, critical infrastructures, and cultural heritage structures. More generally, recent advances in QMEMS technology are progressively closing the performance gap between MEMS-based devices and conventional force-balance and piezoelectric accelerometers. This evolution is enabling a new generation of cost-effective, distributed monitoring systems spanning structural health monitoring (SHM) and urban seismic observatories (USOs), with broad applications in impact-based earthquake early warning (EEW), predictive maintenance, and digital twins. Based on the laboratory experiments, field deployments, and the experience gained from the Italian National Seismic Networks, Urban Seismic Observatories (USOs), and SHM applications, Table 6 summarizes indicative self-noise targets and timing-performance levels for representative seismic and structural monitoring applications.

These two parameters address different aspects of measurement quality: self-noise primarily controls the capability to resolve weak signals, whereas timing accuracy becomes increasingly important when coherent phase relationships among distributed sensing nodes must be preserved. The reported values should therefore be interpreted as application-oriented engineering guidelines rather than strict acceptance thresholds. As illustrated by the sensor noise levels compared in Figure 1, the self-noise values reported in Table 6 should not be interpreted as exclusion thresholds. Sensors with higher self-noise can still provide reliable earthquake recordings when the ground-motion amplitude is sufficiently above the instrumental noise floor, whereas progressively lower self-noise becomes important for resolving weak seismic signals and low-amplitude structural vibrations.

Collectively, these case studies demonstrate that recent advances in low-noise QMEMS technology enable a common sensing platform spanning applications from Operational Modal Analysis of individual structures to permanent Structural Health Monitoring and dense Urban Seismic Observatories, thereby strengthening the convergence between structural engineering and engineering seismology.

## 5. Concluding Remarks

This work presented the laboratory characterization and field validation of the Quartz MEMS Epson M-A370 accelerometer, complementing these results with the extensive experience gained from the previous-generation M-A352 sensor. Rather than focusing solely on the sensing element, the study experimentally assessed a complete QMEMS-based sensing platform integrating ultra-low-noise accelerometers, precise timing synchronization, deterministic data acquisition, embedded processing, and edge-computing capabilities for seismological and Structural Health Monitoring (SHM) applications. The platform was evaluated through a comprehensive multi-scale framework encompassing laboratory characterization, controlled shake-table experiments, and temporary and permanent field deployments under real environmental conditions. Collectively, these investigations demonstrate the versatility of the platform and support its applicability across a broad range of seismic and structural monitoring scenarios.

The comparison with conventional MEMS devices, piezoelectric accelerometers, and reference force-balance instruments shows that recent advances in Quartz MEMS technology have substantially reduced the historical performance gap between MEMS and high-end seismic instrumentation. Experimental observations indicate that self-noise amplitude spectral densities below 1 μg/√Hz, preferably below 0.5 μg/√Hz and, for the most demanding applications, approaching or below 0.1 μg/√Hz, represent practical performance targets for weak-motion seismology, Operational Modal Analysis (OMA), and long-term Structural Health Monitoring (SHM) under weak ambient excitation or in very quiet environments. However, these values should not be interpreted as exclusion thresholds, since accelerometers with higher self-noise can still provide reliable earthquake recordings when the ground-motion amplitude is sufficiently above the instrumental noise floor.

The M-A370, with a self-noise approaching 0.02 μg/√Hz in the 1–10 Hz frequency band, further extends the use of MEMS technology to applications traditionally relying on force-balance accelerometers, IEPE piezoelectric accelerometers and, in some cases, wideband seismometers. While accelerometer self-noise primarily governs the capability to resolve weak seismic and structural signals, timing accuracy becomes increasingly important when coherent phase relationships among distributed sensing nodes are required, particularly for mode-shape identification, wave-propagation analysis, and distributed structural monitoring. Accordingly, both self-noise and timing performance should be evaluated in relation to the specific application, frequency range, expected signal amplitude, and measurement objectives.

The timing experiments showed that the Smart Sensor Box provides high-accuracy synchronization under GNSS-disciplined operation. Under GNSS-denied holdover following initial GNSS synchronization, the current VCXO-based architecture maintains adequate timing stability for temporary distributed OMA and SHM surveys lasting approximately 1–2 h. Although preliminary and limited to a single prototype tested under ambient laboratory conditions, the TCVCXO measurements demonstrated substantially improved holdover performance compared with the currently adopted VCXO, with timing offsets of only 22–51 μs during the first 2–3 h and an accumulated offset remaining below 200 μs after 6 h. These results support its implementation in the next hardware revision of the Smart Sensor Box. This upgrade is expected to reduce timing drift and may enable periodic GNSS resynchronization rather than continuous GNSS disciplining, potentially reducing power consumption. Its stability under temperature variations will be assessed through the planned controlled thermal-validation campaign.

The representative applications described in this study—including Urban Seismic Observatories, bridge monitoring, cultural heritage structures, ordinary buildings, and the integrated monitoring framework developed for the Campi Flegrei area—illustrate how a common sensing architecture can support weak- and strong-motion seismology, OMA, continuous SHM, rapid post-earthquake assessment, and emerging impact-based EEW. These experiences highlight the growing convergence between seismology and structural engineering, whereby dense seismic observations and structural response measurements can be integrated within a unified monitoring framework.

Within this broader convergence, the literature describes several distributed monitoring architectures that provide useful but only partially comparable reference points for the present platform [20,34]. Representative examples include the Community Seismic Network for dense urban seismic monitoring [16] and synchronized wireless smart-sensor networks developed for vibration-based SHM [40]. Networks based on conventional low-cost MEMS devices enable dense, low-power deployments but may be less suitable for weak-motion and high-quality modal-identification applications because of higher sensor self-noise, timing variability, and potentially reduced long-term stability. Conversely, monitoring systems based on force-balance or piezoelectric IEPE sensors, as well as other engineering-grade instrumentation, provide well-established metrological performance but generally involve higher instrumentation costs, more complex installation, and reduced scalability.

Against this background, the developed QMEMS-based Smart Sensor Box occupies an intermediate position in terms of technical performance and potential system-level cost-effectiveness, combining the accelerometer’s ultra-low self-noise and high dynamic range with precise timing, multisensor integration, local storage, and edge processing within a modular distributed architecture. The use of the Smart Sensor Boxes as autonomous nodes reduces cabling and centralized acquisition requirements, facilitating rapid deployment on spatially extended structures and minimally invasive installation on cultural heritage assets. Complementing these deployment advantages, local processing capabilities align the platform with the growing adoption of edge computing in next-generation monitoring networks. Specifically, local processing reduces communication bandwidth requirements, preserves acquired data during network interruptions, and provides the computational basis for rapid alert generation from key strong-motion parameters, AI-assisted event classification, real-time ground-motion estimation, impact-based EEW, and, where appropriate, the local triggering of safety actuators.

However, these advantages are accompanied by inherent trade-offs. The QMEMS-based Smart Sensor Box is more expensive than conventional low-cost MEMS devices, while its onboard architecture—including a dedicated timing unit and GNSS receiver, embedded computer, communication interfaces, and local storage—increases hardware complexity and overall power demand, despite the use of low-power components and energy-efficient operating strategies. At the system level, the deployment advantages described above may nevertheless reduce installation complexity and lifecycle costs compared with conventional accelerometric networks or distributed SHM systems employing force-balance or IEPE piezoelectric accelerometers.

The QMEMS-based Smart Sensor Box should therefore not be regarded as a universal replacement for existing monitoring solutions; its suitability should instead be evaluated in relation to the specific monitoring strategy, required noise floor, frequency range, timing accuracy, deployment density and constraints, autonomy, communication environment, and overall lifecycle cost.

## Figures and Tables

**Table 1:** Comparison of representative force-balance (FBA), MEMS, and QMEMS accelerometers for seismic monitoring and Structural Health Monitoring applications. Some of the listed sensors were employed in the experimental activities presented in this work. Specifications are taken from the corresponding manufacturers’ datasheets.

Company/Model	Technology/Axes	Output	Full-Scale Range (g)	Sensitivity/Scale Factor	Typical Self-Noise Density (µg/√Hz)	Frequency Response/Band Width	Dynamic Range (dB)	Representative Applications
Kinemetrics, Inc. (Pasadena, CA, USA)/EpiSensor ES-T	Force-balance accelerometer (FBA) 3-axis	Analog (selectable)	±0.25 to ±4	up to 10,000 (mV/g)	0.06 @1 Hz (±0.25 g range)	DC–200 Hz	155	Strong-motion seismology; structural monitoring; EEW
Nanometrics Inc. (Kanata, ON, Canada)/Titan	Force-balance accelerometer (FBA) 3-axis	Analog differential (40 Vpp)	±0.25 to ±4	Depends on the selected gain (full-scale range).	comparable to some BB seismometers	DC–430 Hz (–3 dB)	166	Weak- and strong-motion seismology; high-resolution structural monitoring
Güralp Systems Ltd. (Aldermaston, Reading, UK)/Fortimus	Force-feedback digital accelerometer + integrated digitiser 3-axis	Digital 24-bit	±0.5 to ±4	Depends on the selected gain (full-scale range).	self-noise below NHNM (>0.07 Hz) and AHNM (DC–100 Hz)	DC–315 Hz	>165	Permanent seismic monitoring; local EEW; SHM
Lunitek S.r.l. (Sarzana, La Spezia, Italy)/Triton FB160	Force-balance accelerometer (FBA) + integrated digitiser 3-axis	Digital 24-bit	±0.5 to ±4	10 V/g @ 1g Res. 0.12 µg @ 1g		DC-80 Hz (opt. DC-200 Hz)	160 (digitizer 136 @100 sps)	Permanent seismic and structural monitoring
Seiko Epson Corporation (Suwa-gun, Nagano, Japan)/M-A370AD10	QMEMS (quartz resonant DETF); 3-axis	Digital 32-bit (SPI/UART)	±10	0.06 (µg/LSB)	0.02–0.04 (1–10 Hz)	DC–210 Hz (selectable integrated LPF)	~170	Weak- and strong-motion seismology; OMA; SHM; USNs; EEW
Seiko Epson M-A352AD10	QMEMS (quartz resonant DETF) 3-axis	Digital 32-bit (SPI/UART)	±15	0.06 (µg/LSB)	0.2–0.7 (0.5–30 Hz)	DC–460 Hz (−6 dB, selectable)	~148	Strong-motion seismology; OMA; SHM; USNs; EEW
Analog Devices, Inc. (Wilmington, MA, USA)/ADXL355	Capacitive MEMS, digital 3-axis	Digital 20-bit (SPI/I^2^C)	±2 to ±8	3.9 µg/LSB (@±2 g)	22.5	Programmable LPF passband 0.977–1000 Hz	~90 (±2 g)	Low-cost seismic and vibration monitoring; USNs; strong-motion recording; EEW
TE Connectivity–MEAS (Aliso Viejo, CA, USA)/4630A	Gas-damped MEMS DC triaxial, analog 3-axis	Analog differential	±2	1000 mV/g (@±2 g)	2 RMS (spectral, @±2 g)	0–150 Hz (±5%); 0–400 Hz (±1 dB)	~95–98	Low-frequency vibration monitoring; SHM
Safran Sensing Technologies (Yverdon-les-bains, Switzerland SA) SI1003	Capacitive MEMS, analog (strong-motion class B) 1-axis	Analog differential	±3	900 (mV/g)	0.7–0.9	Freq. response +3 dB: 450–550 Hz	108.5 (0.1–100 Hz)	Strong-motion monitoring; SHM; OMA; industrial vibration monitoring; and EEW
Safran Sensing Technologies VS1002	Capacitive MEMS, analog (high-end vibration) 1-axis	Analog differential	±2	1350 (mV/g)	7	Freq. response in-band (typ): DC to ~700 Hz	86 (0.1–100 Hz)	High-resolution vibration and structural monitoring

Note: Dynamic-range and self-noise values may refer to different full-scale settings, frequency bands, and measurement conditions and are therefore intended for indicative rather than strictly metrological comparison. The achievable system dynamic range depends on the acquisition chain, including sensor configuration, signal conditioning, ADC resolution, and processing electronics.

**Table 2:** Comparison of representative IEPE (ICP^®^) accelerometers commonly used for structural dynamics, Operational Modal Analysis (OMA), Structural Health Monitoring (SHM), and laboratory testing. Some of the listed sensors were employed in the experimental activities presented in this study. Specifications were obtained from the corresponding manufacturers’ datasheets, whereas dynamic-range values were estimated as described in the table note.

Company/Model	Technology/Axes	Output	Full-Scale Range (g)	Sensitivity/Scale Factor	Noise Density (µg/√Hz)	Bandwidth/Response	Dynamic Range (dB)	Representative Applications
PCB Piezotronics, Inc. (Depew, NY, USA)/ 393B31	Silicon MEMS, analog 1-axis	Analog IEPE (ICP^®^)	±0.5	10 V/g	0.06 @1 Hz 0.01 @10 Hz 0.004 @100 Hz	0.1–200 Hz	~136	High-resolution, low-frequency structural vibration monitoring; OMA and SHM
PCB Piezotronics 393B04	Piezoelectric (ICP^®^), seismic 1-axis	Analog IEPE (ICP^®^)	±5	1000 mV/g	Spectral noise: 0.30 @1 Hz 0.10 @10 Hz 0.04 @100 Hz	0.06–450 Hz (±5%)	~136	Low-amplitude structural vibration monitoring; OMA and SHM
PCB Piezotronics 393B12	Piezoelectric (ICP^®^), seismic (high sensitivity) 1-axis	Analog IEPE (ICP^®^)	±0.5	10,000 mV/g	Spectral noise: 1.30 @1 Hz 0.32 @10 Hz 0.13 @100 Hz 0.10 @1 kHz	0.15–1000 Hz (±5%)	~106	High-sensitivity structural vibration monitoring; OMA and SHM
PCB Piezotronics 333B50	Piezoelectric (ICP^®^), modal-structural 1-axis	Analog IEPE (ICP^®^)	±5	1000 mV/g	Spectral noise: 15 @1 Hz 3.8 @10 Hz 1.1 @100 Hz 0.4 @1 kHz	0.5–3000 Hz (±5%)	~105	Modal testing and structural vibration monitoring; OMA and SHM
PCB Piezotronics 356A17	Piezoelectric (ICP^®^), structural 3-axis	Analog IEPE (ICP^®^)	±10	500 mV/g	Spectral noise: 18 @1 Hz 6 @10 Hz 2 @100 Hz 0.7 @1 kHz	0.5–3000 Hz (±5%)	~107	Triaxial structural testing and vibration monitoring; OMA and SHM

Note: Dynamic-range values were estimated over the 0.5–30 Hz frequency band, assuming white noise equal to the spectral noise density specified at 10 Hz and a full-scale sinusoidal RMS amplitude (FS/√2). However, the actual dynamic range depends on the complete acquisition chain, including IEPE signal conditioning and ADC resolution.

**Table 3:** Indicative frequency ranges and inter-node synchronization accuracies for representative seismic and structural monitoring applications.

Application	Indicative Frequency Range of Interest (Hz)	Indicative Relative Timing Accuracy
Ambient-vibration monitoring/basic OMA	0.2–20	0.5–1 ms
Distributed OMA/mode-shape identification	0.1–30	0.1–1 ms; ≤0.1 ms desirable for high-quality, phase-consistent mode-shape identification
Vibration-based Structural Health Monitoring (SHM)	0.1–50	0.1–1 ms; ≤0.1 ms desirable for phase-sensitive distributed analyses
Local seismic arrays	0.1–50	0.1–1 ms
Urban seismic networks	0.1–40	0.1–2 ms; sub-ms desirable for coherent network analyses and event characterization

**Table 4:** Summary of measured timing performance under GNSS-disciplined and GNSS-denied holdover conditions. Laboratory VCXO and TCVCXO measurements characterize the timing-unit performance relative to the GNSS-derived PPS, whereas CT02–CT04 measurements quantify the relative timing offset between two complete Smart Sensor Boxes. Equivalent timing offsets are expressed in samples at 200 and 1000 Hz.

Timing Architecture/ Operating Condition	Holdover Duration	Measured Timing Offset (μs)	Equivalent Sampling Offset at 200 Hz (Samples)	Equivalent Sampling Offset at 1000 Hz (Samples)
VCXO—Laboratory GNSS-disciplined	—	14.2	0.00284	0.0142
VCXO—Laboratory in holdover	4.4 h	±80	±0.016	±0.080
VCXO—(CT02-CT04) both GNSS-disciplined	—	9.1	0.00182	0.0091
VCXO—(CT02-CT04) in holdover	1 h	160	0.032	0.16
VCXO—(CT02-CT04) in holdover	2-3 h	300–450	0.060–0.090	0.300–0.450
VCXO—(CT02-CT04) in holdover	6 h	~300	~0.060	~0.300
VCXO—(CT02-CT04) in holdover	24 h	~9000	~1.80	~9.00
TCVCXO—Laboratory GNSS-disciplined	—	7.8	0.00156	0.0078
TCVCXO—Laboratory in holdover	2 h	22	0.0044	0.022
TCVCXO—Laboratory in holdover	3 h	51	0.0102	0.051
TCVCXO—Laboratory in holdover	6 h	194	0.0388	0.194
TCVCXO—Laboratory in holdover	24 h	1700	0.34	1.7

Note: The relative timing offset measured during VCXO holdover exhibited a non-monotonic evolution. Accordingly, the values reported at the indicated elapsed times represent observed offsets that are not necessarily cumulative or monotonic; the approximately 300 μs offset at 6 h may therefore be lower than the 300–450 μs range observed after 2–3 h.

**Table 5:** Modal parameters identified by Operational Modal Analysis for the first three monitored buildings (ED001–ED003), including modal frequency, damping ratio, and complexity index.

Building	Mode	Frequency (Hz)	Damping (%)	Complexity (%)
ED001	Flexural	2.073	1.205	0.109
	Flexural	2.200	0.974	0.259
	Torsional	2.924	1.077	0.015
ED002	Flexural	1.941	1.330	1.094
	Flexural	2.090	1.697	0.250
	Torsional	2.659	1.275	0.296
ED003	Flexural	2.299	1.786	0.026
	Flexural	2.499	1.171	0.121
	Torsional	3.365	1.618	0.100

**Table 6:** Indicative self-noise targets and timing-performance levels for representative seismic and structural monitoring applications. The reported values are intended as application-oriented engineering guidelines rather than universal acceptance thresholds.

Representative Application	Indicative Self-Noise Density Target	Indicative Inter-Node Timing Performance
Strong-motion monitoring/EEW	Self-noise is generally less critical; sensors with >1 µg/√Hz may be adequate when earthquake-induced ground motion is sufficiently above the instrumental noise floor	≤1 ms generally adequate; sub-ms desirable for phase-consistent multi-station analyses
Weak-motion seismic monitoring	<1 μg/√Hz; <0.5 μg/√Hz preferable for low-amplitude signals	Sub-ms synchronization desirable for distributed observations
Urban Seismic Networks/Urban Seismic Observatories	<1 μg/√Hz; <0.5 μg/√Hz preferable for weak motion observations	0.1–2 ms; sub-ms desirable for phase-consistent network analyses
Ambient-vibration monitoring/basic OMA	<1 µg/√Hz; ≤0.5 µg/√Hz preferable	0.5–1 ms generally adequate; less stringent when all channels share a common acquisition clock
Distributed OMA/mode-shape identification	<0.5 μg/√Hz preferable; approaching or below 0.1 μg/√Hz desirable in very quiet environments	0.1–1 ms; ≤100 µs desirable for high-quality, phase-consistent mode-shape identification
High-sensitivity/long-term SHM	<0.5 μg/√Hz; approaching or below 0.1 μg/√Hz desirable under very weak excitation	0.1–0.5 ms generally adequate; ≤100 µs desirable for phase-sensitive distributed analyses; otherwise application-dependent

Note: Self-noise is frequency dependent, and the actual requirements depend on signal amplitude, environmental noise, sensor configuration, operating conditions, and analysis objectives.

## Data Availability

The data presented in this study are available on request from the corresponding author.
